# Synthesis of PdS_x_-Mediated Polydymite Heteronanorods and Their Long-Range Activation for Enhanced Water Electroreduction

**DOI:** 10.34133/2019/8078549

**Published:** 2019-08-18

**Authors:** Qiang Gao, Rui Wu, Yang Liu, Ya-Rong Zheng, Yi Li, Li-Mei Shang, Yi-Ming Ju, Chao Gu, Xu-Sheng Zheng, Jian-Wei Liu, Jun-Fa Zhu, Min-Rui Gao, Shu-Hong Yu

**Affiliations:** ^1^Division of Nanomaterials & Chemistry, Hefei National Laboratory for Physical Sciences at the Microscale, CAS Center for Excellence in Nanoscience, Hefei Science Center of CAS, Collaborative Innovation Center of Suzhou Nano Science and Technology, Department of Chemistry, University of Science and Technology of China, Hefei 230026, China; ^2^National Synchrotron Radiation Laboratory, University of Science and Technology of China, Hefei 230026, China; ^3^Dalian National Laboratory for Clean Energy, Dalian 116023, China

## Abstract

Material interfaces permit electron transfer that modulates the electronic structure and surface properties of catalysts, leading to radically enhanced rates for many important reactions. Unlike conventional thoughts, the nanoscale interfacial interactions have been recently envisioned to be able to affect the reactivity of catalysts far from the interface. However, demonstration of such unlocalized alterations in existing interfacial materials is rare, impeding the development of new catalysts. We report the observation of unprecedented long-range activation of polydymite Ni_3_S_4_ nanorods through the interfacial interaction created by PdS_x_ nanodots (dot-on-rod structure) for high-performance water catalytic electroreduction. Experimental results show that this local interaction can activate Ni_3_S_4_ rods with length even up to 25 nanometers due to the tailored surface electronic structure. We anticipate that the long-range effect described here may be also applicable to other interfacial material systems, which will aid the development of newly advanced catalysts for modern energy devices.

## 1. Introduction

It has been almost 46 years since the proposition of the* hydrogen economy* concept, which depicted a clean, safe, and sustainable alternative to the current hydrocarbon economy [1]. Recent research has led to many advances towards this blueprint, but large-scale hydrogen production through electrocatalysis at low overpotentials (*η*) remains a challenge [2]. Although expensive precious metals such as platinum capable of catalyzing the hydrogen evolution reaction (HER) at fast rates are known, the reliable and scalable electrolyzers require low-cost and efficient catalysts based on geologically abundant elements [3, 4]. In conventional heterogeneous catalysis, an intuitive and commonly used method towards better catalysts is to couple transition metal nanoparticles with oxide or carbon supports, which creates the so-called ‘strong metal-support interactions' that modulate the electronic structure and surface properties of catalytic materials, leading to enhanced performances [5–14]. This interaction-induced phenomena has been extensively studied since being discovered by Tauster et al. in 1970s [14], which is now understood to be due to the electron transfer across the formed interfaces [7]. Lykhach and coworkers have quantified the electron transfer experimentally on a Pt-CeO_2_ catalyst and observed a characteristic particle size dependence [7]. Commonly, such interaction is thought to be localized at the subnanometre scale, suggesting that only reactive sites in the immediate vicinity of the interface are influenced [5, 15]. Very recently, Suchorski and coworkers' study with Pd-metal oxide catalysts, however, demonstrated that the interfacial interaction can activate the CO oxidation energetics of Pd sites thousands of nanometers away from the interface [5]. In recent years, material interface engineering has also led to substantial advances in designing electrocatalysts via the interface-created structural perturbations [16–18]. Nonetheless, whether the long-range activation phenomenon exists in these interfacial electrocatalyst systems, for example, the heterostructures, remains unknown and needs to be clarified experimentally, which could be complementary for understanding the interface-induced enhancement behavior and yield better performing HER electrocatalysts.

In nature, hydrogenases are able to catalyze the HER at potentials close to its thermodynamic value (2H^+^ + 2e^−^ → H_2_; 0-0.059 × pH, V versus normal H_2_ electrode at 298 K), despite using cheap metals, such as nickel, iron, and molybdenum, as active sites [19]. This leads to investigations of inorganic analogues or complexes that mimic such active centers as catalysts for HER, for example, the representative molybdenite MoS_2_ [20–22]. Besides molybdenum, chalcogenides of many other elements, such as iron [23, 24], cobalt [25–28], and nickel [23, 24, 29], have also shown significant potentials in HER electrocatalysis. Of these, nickel sulfides are particularly intriguing because they can form various phases such as NiS, NiS_2_, Ni_3_S_2_, Ni_3_S_4_, Ni_7_S_6_, and Ni_9_S_8_ that offer diverse properties [30], and because they had demonstrated good uses as electrode materials for Li-ion batteries [31–33], supercapacitors [34–36], and catalysts for hydrodesulfurization reactions [37]. Previous works were focused primarily on heazlewoodite Ni_3_S_2_ [29, 38], which holds promise for electrocatalysis of oxygen reduction [39] and hydrogen evolution [29]; but studies on other nickel sulfides are comparatively rare, leaving their catalytic properties largely unexplored. For example, polydymite Ni_3_S_4_ is a common mineral existed in ores, which crystallizes in cubic spinel structure with Ni^2+^/Ni^3+^ couple [40, 41]. Although interesting structure, methods of synthesizing nanostructured Ni_3_S_4_ often lead to significant phase impurity [42], which hampers the technological exploitation of Ni_3_S_4_ as a HER catalyst, even though both nickel and sulfur are essential to hydrogenases [19]. Yet engineering these metal chalcogenides, for example, the activation of single phase Ni_3_S_4_ via interfacial interactions, that aims for high-performance HER catalysis is even more challenging.

Herein, we report the synthesis of high-pure Ni_3_S_4_ nanorods mediated with nanoparticulate PdS_x_ on the tip of each nanorod, where the resulting PdS_x_-Ni_3_S_4_ interface shows an unprecedented long-range effect on the reactivity of Ni_3_S_4_ in water catalytic electroreduction. PdS_x_ was the material of choice because it is conductive and chemically robust under harsh conditions such as low pH and high temperature [43]. We reveal that such interfacial interaction enables the activation of Ni_3_S_4_ nanorods with length up to 25 nm, making the PdS_x_-Ni_3_S_4_ a highly active and stable HER catalyst. We understand the interaction-induced enhancement based on a range of experimental investigations and propose that remarkable charge transfer across the PdS_x_-Ni_3_S_4_ interface enables surface structural optimization of Ni_3_S_4_ nanorods, giving rise to the catalytic promotions. These findings may be potentially applied to other material systems and lead to broader libraries of interfacial catalysts for reactions beyond H_2_ evolution.

## 2. Results

### 2.1. Synthesis and Structural Characterizations of the PdS_x_-*Ni*_3_*S*_4_ Heteronanorods

**Figure 1:**
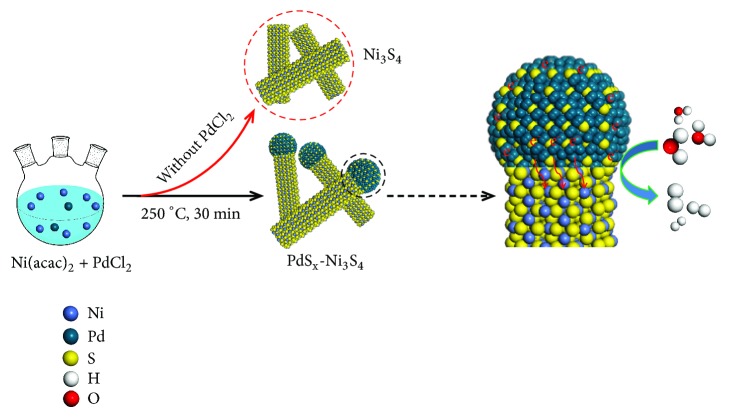
*The synthesis of PdS*_*x*_ *-Ni*_3_ *S*_4_ *heteronanorods.* Schematic illustration of the synthesis of the PdS_x_-Ni_3_S_4_ heteronanorods, showing PdS_x_ mediates the electronic structure of polydymite Ni_3_S_4_. Blue, yellow, and cyan balls correspond to Ni, S, and Pd atoms, respectively.

**Figure 2:**
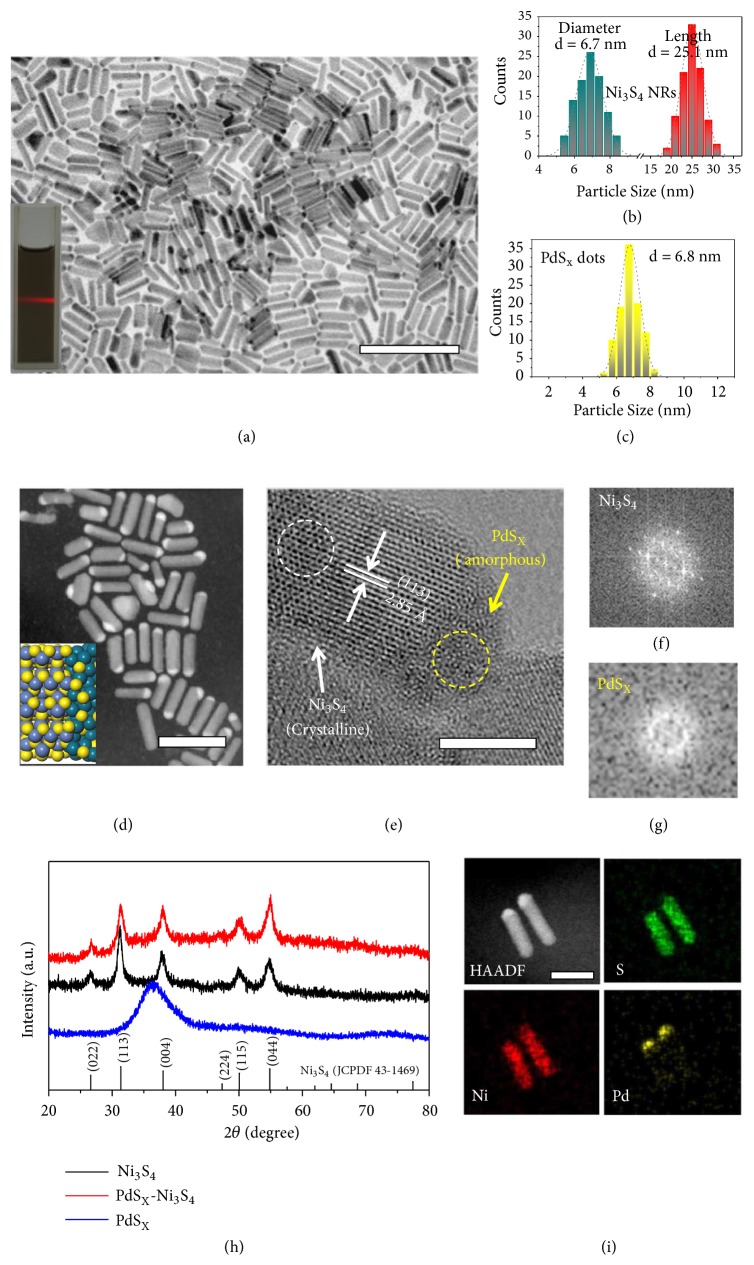
*Characterizations of the PdS*_*x*_ *-Ni*_3_ *S*_4_ *heteronanorods.* (a) TEM image of synthesized PdS_x_-Ni_3_S_4_ heteronanorods. Scale bar, 50 nm. A digital image of PdS_x_-Ni_3_S_4_ heteronanorods in hexane is shown as inset. (b and c) Histograms of PdS_x_-Ni_3_S_4_ heteronanorods showing the size of Ni_3_S_4_ nanorods and PdS_x_ dots, respectively. (d) HAADF-STEM image of PdS_x_-Ni_3_S_4_ heteronanorods. Scale bar, 20 nm. Inset gives the crystal structure of PdS_x_-Ni_3_S_4_. Blue, yellow, and cyan balls correspond to Ni, S, and Pd atoms, respectively. (e) HRTEM image of a typical PdS_x_-Ni_3_S_4_ heteronanorod. Scale bar, 5 nm. (f and g) The FFT patterns taken from the regions marked by white and yellow dashed circles of (e), featuring the crystalline Ni_3_S_4_ and amorphous PdS_x_, respectively. (h) XRD patterns of PdS_x_-Ni_3_S_4_ heteronanorods, pure Ni_3_S_4_, and PdS_x_. (i) HAADF image and STEM elemental mapping of PdS_x_-Ni_3_S_4_ heteronanorods. Scale bar, 20 nm.

We achieved the synthesis of well-defined, pure-phase Ni_3_S_4_ nanorods functionalized with nanoparticulate PdS_x_ terminations (i.e., dot-on-rod structure) by consecutive thermolysis of corresponding metal precursors, as illustrated schematically in Figure 1. Briefly, [Ni(acac)_2_] (acac = acetylacetonate) and PdCl_2_ were mixed in a solution containing 1-dodecanethiol (DDT) and oleylamine (OAm), which was then heated to 250°C and maintained for 30 minutes. In the synthesis of the PdS_x_-Ni_3_S_4_ colloidal heteronanorods, DDT acts as the sulfur source, while OAm acts as both the solvent and stabilizer. Transmission electron microscopy (TEM) image of the as-synthesized sample reveals uniform ‘dot-on-rod'-like structures with the dot size of ~6.8 nm, as well as the rod length and diameter of ~25.1 nm and ~6.7 nm, respectively (Figures 2(a)–2(c)). High-angle annular dark-field scanning TEM (HAADF-STEM, Figure 2(d) and inset) image clearly shows the ‘dot-on-rod' heterostructure that corresponds to PdS_x_ (bright) and Ni_3_S_4_ (gray), respectively, owing to the* Z*^2^-dependent contrast (*Z* is the atomic number). Studies with high-resolution TEM (HRTEM, Figure 2(e)) demonstrate good crystallinity of Ni_3_S_4_ nanorods with resolved lattice fringe of (113) plane, which are free from any secondary phases, whereas the PdS_x_ dot presents as unexpected amorphous phase (Figures 2(e), [Supplementary-material supplementary-material-1], and [Supplementary-material supplementary-material-1]). The fast Fourier transform (FFT) patterns taken from the dashed circles again evidence the crystalline (Figure 2(f)) and amorphous (Figure 2(g)) structures, respectively. We further confirmed this through X-ray diffraction (XRD) studies of the product (Figure 2(h), red curve), in which the strong diffraction peaks are assigned to cubic Ni_3_S_4_ with spinel structure (JCPDS No. 43-1469). Our XRD studies show almost negligible peak at 37.5° resulting from the amorphous PdS_x_ (Figure 2(h), red and blue curves) [44], consistent with the above observations. Energy-dispersive X-ray spectroscopy (EDS) confirms the expected chemical elements although it picked up Cu and C signals from the TEM grid ([Supplementary-material supplementary-material-1]). STEM elemental mapping of the PdS_x_-Ni_3_S_4_ sample shows Pd-rich dots and Ni-rich rods with S enrichment in the whole structure (Figures 2(i) and [Supplementary-material supplementary-material-1]), matching well with our EDS line scan that passes through the central axis of a typical heteronanorod ([Supplementary-material supplementary-material-1]). We have also determined that the molar content of PdS_x_ in PdS_x_-Ni_3_S_4_ heteronanorods is about 10.5% based on the inductively coupled plasma mass spectrometer (ICP-MS) studies, which agrees with the ~10% value measured by EDS. Together, these results support that we have succeeded in synthesizing the new, uniform, and high-pure PdS_x_-Ni_3_S_4_ heteronanorods.

We designed and conducted a series of control experiments to explore the formation of PdS_x_-Ni_3_S_4_ heteronanorods. By varying the amount of 1-dodecanethiol we can effectively tune the length of Ni_3_S_4_ nanorods in the ‘dot-on-rod' structure. For example, adding 0.25 mL 1-dodecanethiol into the reaction system results in Ni_3_S_4_ nanorods with length of ~15 nm, which substantially increases to ~25.1 and ~34.8 nm after addition of 0.5 and 0.6 mL 1-dodecanethiol, respectively ([Supplementary-material supplementary-material-1]). However, no large growth for the PdS_x_ nanoparticles was seen in these experiments. Further, increasing the amount of 1-dodecanethiol yields PdS_x_-Ni_3_S_4_ heterostructure with rod coarsening, while a lower addition of 0.1 mL gives spherical nanoparticles instead of nanorods ([Supplementary-material supplementary-material-1]). These observations indicate that the size and shape of Ni_3_S_4_ are controlled via 1-dodecanethiol. As to PdS_x_, we found that its growth shows a pronounced temperature dependence. Reaction at temperature of 230°C results in dominant Ni_3_S_4_ nanorods without PdS_x_, suggesting the high formation energy of PdS_x_ species. But too much thermal input (e.g., 270°C) induces isotropic growth, forming nonuniform nanoparticles ([Supplementary-material supplementary-material-1]). We determined the appropriate temperature for synthesizing PdS_x_-Ni_3_S_4_ heteronanorods is 250°C. Moreover, Pd:Ni molar ratio in the reaction system is also critical and the best value was uncovered to be 1:4. Deviating from this value will make the product form irregular or nonuniform structures ([Supplementary-material supplementary-material-1]). We carefully examined the samples at different stages during the synthesis by TEM to probe the evolutionary process of PdS_x_-Ni_3_S_4_ heteronanorods ([Supplementary-material supplementary-material-1]). Rod-like product appeared when the mixture reached 250°C, but without PdS_x_ terminations. At early stage (5 min), particulate PdS_x_ started to emerge at the tip of each nanorod, which grew in size as the reaction proceeded, forming optimal PdS_x_-Ni_3_S_4_ heteronanorods at 30 min. These observations are somewhat similar to the synthesis of anisotropically phase-segregated PdS_x_-Co_9_S_8_ and PdS_x_-Co_9_S_8_-PdS_x_ nanoacorns reported previously by Teranishi and coworkers [44, 45]. We further note that if no Pd precursor was provided, pure Ni_3_S_4_ nanorods would result (Figures [Supplementary-material supplementary-material-1] and [Supplementary-material supplementary-material-1]); if without Ni addition, nanoparticulate PdS_x_ would form, but at a higher temperature of 300°C (Figures [Supplementary-material supplementary-material-1]-[Supplementary-material supplementary-material-1]). Lower temperature such as 250°C gives yellow Pd precursor with unknown phase (Figures [Supplementary-material supplementary-material-1]-[Supplementary-material supplementary-material-1]). However, in the PdS_x_-Ni_3_S_4_ growth system, the preformed Ni_3_S_4_ nanorods can allow heterogeneous nucleation of PdS_x_ on their tips to substantially lower the nucleation barrier, which permits the formation of PdS_x_-Ni_3_S_4_ at lower temperature of mere 250°C. Because of the deficient energy for crystallization, the PdS_x_ nanodots on the tips show the amorphous nature.

### 2.2. Interactions between PdS_x_ Nanoparticles and *Ni*_3_*S*_4_ Nanorods

**Figure 3:**
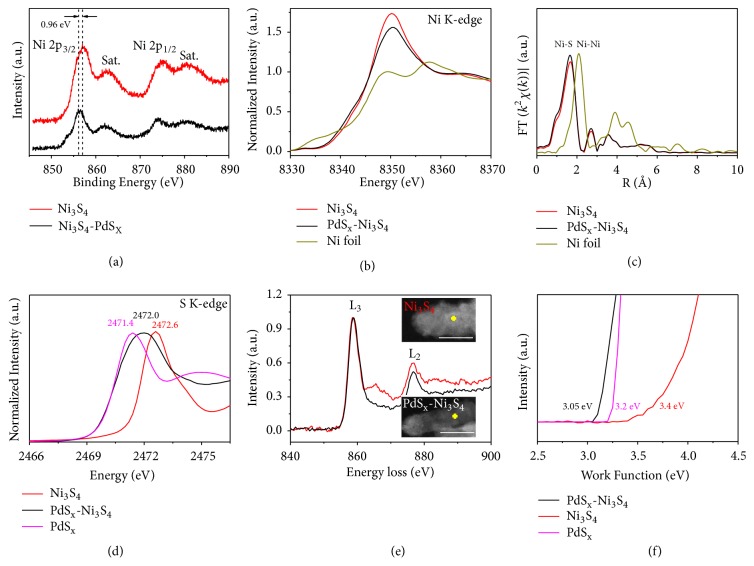
*Electronic structure modulation in PdS*_*x*_ *-Ni*_3_ *S*_4_ *heteronanorods.* (a) Ni 2*p* XPS spectra of PdS_x_-Ni_3_S_4_ and pure Ni_3_S_4_, showing a decrease of ~0.96 eV after coupling Ni_3_S_4_ with PdS_x_. (b) Ni* K*-edge XANES spectra of PdS_x_-Ni_3_S_4_, Ni_3_S_4_, and Ni foil reference. (c) EXAFS Fourier-transformed* k*^3^-weighted* χ*(*k*) function spectra of PdS_x_-Ni_3_S_4_, Ni_3_S_4_ and Ni foil reference. (d) S* K*-edge XANES spectra of PdS_x_-Ni_3_S_4_, pure Ni_3_S_4_, and PdS_x_. (e) EELS spectra of PdS_x_-Ni_3_S_4_ and Ni_3_S_4_ at Ni* K*-edge. Insets show the representative positions of EELS acquisition. Scale bars, 10 nm. (f) Ultraviolet photoelectron spectra of PdS_x_-Ni_3_S_4_, pure Ni_3_S_4_, and PdS_x_.

The electronic interactions between PdS_x_ nanoparticles and Ni_3_S_4_ nanorods were comprehensively investigated via multiple characterization techniques (Figures 3 and [Supplementary-material supplementary-material-1]). X-ray photoelectron spectroscopy (XPS) measurements show that the binding energy of Ni 2*p* core levels markedly decreases by ~0.96 eV versus pure Ni_3_S_4_, attributable to charge transfer from PdS_x_ to Ni_3_S_4_ (Figure 3(a)). We highlight that this chemical shift of Ni 2*p* peak is remarkable, which can not solely originate from the local nanoscale PdS_x_-Ni_3_S_4_ interface, but remote surfaces of Ni_3_S_4_ in these heteronanorods should be also affected. Such charge transfer is further confirmed by the shift of absorption edge towards lower energy in X-ray absorption near-edge spectroscopy (XANES) of the Ni* K*-edge (Figure 3(b)). Additionally, the decrease in the white line intensity again verifies that Ni_3_S_4_ accepts electrons from PdS_x_ in the heteronanorods (Figure 3(b)). Figure 3(c) presents Fourier-transformed Ni K-edge extended X-ray absorption fine structure (EXAFS) analysis for studied materials. Features that correspond to the nearest Ni-S coordination (~1.66 Å) are clearly seen for both PdS_x_-Ni_3_S_4_ and pure Ni_3_S_4_, whereas the peak intensity increases for PdS_x_-Ni_3_S_4_. This indicated that the outer shell of the Ni centers in PdS_x_-Ni_3_S_4_ definitely changed compared to that of Ni_3_S_4_ [46]. S* K*-edge XANES spectra in Figure 3(d) show a broad peak at ~2472.0 eV that attributed to characteristic S^2-^, which locates between pure PdS_x_ (~2471.4 eV) and Ni_3_S_4_ (~2472.6 eV), indicating that the S electronic environment was neutralized in PdS_x_-Ni_3_S_4_ heteronanorods because of the electron transfer from PdS_x_ to Ni_3_S_4_ [47–49]. By using electron energy-loss spectroscopy (EELS) in the STEM mode, we measured the L_3_/L_2_ ratio at Ni* L*-edge far away from the rod ends (Figure 3(e) and Insets). Expectedly, we see noticeable larger L_3_/L_2_ ratio for PdS_x_-Ni_3_S_4_ versus pure Ni_3_S_4_, adding further strong support to our finding that long-range electronic modulation can be enabled by the nanoscale interface.

**Figure 4:**
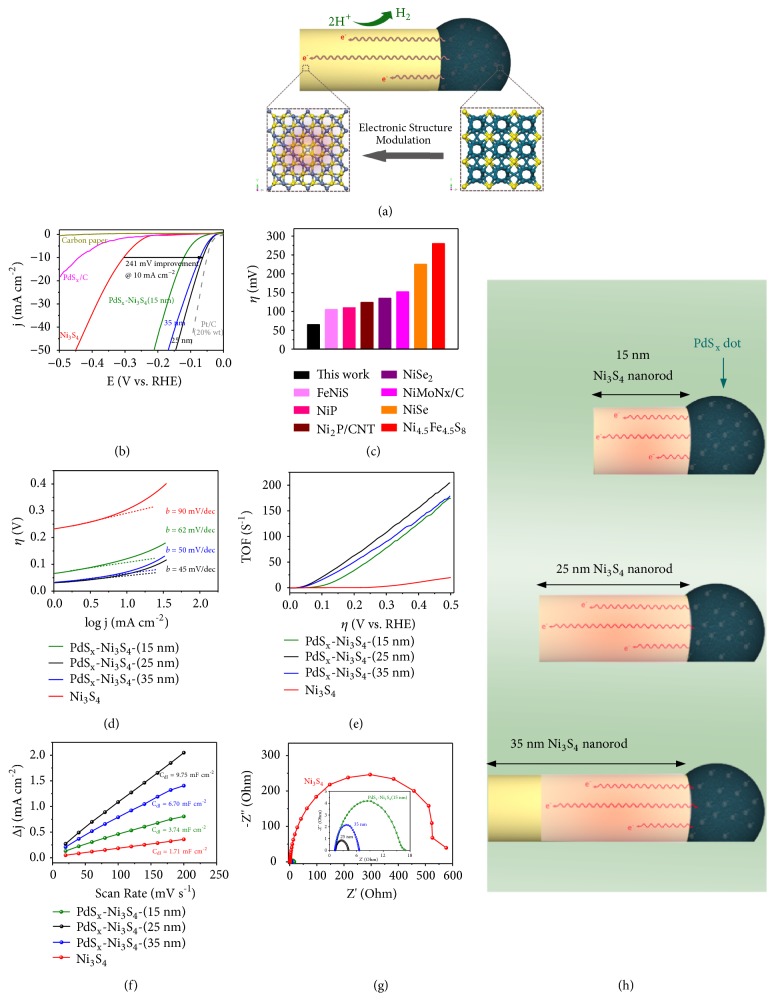
*Water electroreduction evaluation.* (a) Schematic of the long-range activation in PdS_x_-Ni_3_S_4_ heteronanorod. A PdS_x_ dot coupled with Ni_3_S_4_ nanorod to form the strong interfacial interaction that permits remote electronic structure modulation of Ni_3_S_4_, leading to enhanced electrocatalytic properties. (b) Polarization curves for HER of different studied catalysts. Catalyst loading: 1 mg cm^−2^. Sweep rate: 5 mV s^−1^. (c) Comparison of *η* required to yield a current density of 10 mA cm^−2^ on various Ni-based electrocatalysts. (d) Tafel plots for the different catalysts derived from (b). (e) TOF as a function of *η* for different catalysts. (f) Plots showing the extraction of the *C*_dl_ for different catalysts. (g) EIS Nyquist plots of different catalysts. Inset compares Nyquist plots at high-frequency range for PdS_x_-Ni_3_S_4_ heteronanorods with different rod lengths. *Z*′ is the real impedance and -*Z*′′ is the imaginary impedance.* The potential for the Nyquist plot measurement was -0.204 V *versus* RHE.* (h) Schematic of the modulated surface electronic structures of PdS_x_-Ni_3_S_4_ heteronanorod, revealing that the interfacial interaction enable the activation of Ni_3_S_4_ nanorod up to ~25 nm away from the interface.

On the basis of this set of experiments we demonstrate clear long-range impact on surface features in the PdS_x_-Ni_3_S_4_ case as compared with pure Ni_3_S_4_. Furthermore, ultraviolet photoelectron spectroscopy (UPS; Figure 3(f)) measurements reveal that PdS_x_-Ni_3_S_4_ heteronanorods possess lower work function (3.05 eV) relative to metallic PdS_x_ (3.2 eV) and pure Ni_3_S_4_ (3.4 eV). These results offer additional evidence that superior electronic property is gained because of the long-range effect of the nanoscopic interface (Figure 4(a)).

### 2.3. Long-Range Activation in the PdS_x_-*Ni*_3_*S*_4_ Heteronanorods

The long-range activation of Ni_3_S_4_ nanorods via the PdS_x_-Ni_3_S_4_ interfaces was experimentally demonstrated by evaluating their HER activity in N_2_-saturated 0.5 M H_2_SO_4_, with that of pure PdS_x_, Ni_3_S_4_ and Pt/C benchmark for comparison (see Experimental Section). Before electrochemical studies, all of the adsorbed OAm was thoroughly removed by treating the as-synthesized PdS_x_-Ni_3_S_4_ heteronanorods in acetic acid at 70°C for 10 h ([Supplementary-material supplementary-material-1]). We achieved the optimal PdS_x_-Ni_3_S_4_ heterocatalyst for comparative study based on a series of control experiments (Figures [Supplementary-material supplementary-material-1] and [Supplementary-material supplementary-material-1]). Figure 4(b) reveals that the background HER current from the carbon paper support is featureless, while the same cathodic sweep of PdS_x_-Ni_3_S_4_ heteronanorods (~25 nm) exhibits a sharp current jump at about -20 mV versus reversible hydrogen electrode (RHE), accounting for the catalytic HER. In contrast, pure Ni_3_S_4_ starts the HER at larger *η* of 120 mV, whereas free PdS_x_ nanoparticles offer negligible HER activity. At a current density of 10 mA cm^−2^, the recorded *η* for PdS_x_-Ni_3_S_4_ was mere 63 mV versus greatly larger *η* of 304 mV for pure Ni_3_S_4_ (Figure 4(b)). These results clearly reveal that Ni_3_S_4_ nanorods are inherently activated through coupling with PdS_x_ for superior HER energetics, exceeding previously reported performances of other Ni-based HER catalysts (Figures 4(c) and [Supplementary-material supplementary-material-1]). Steady-state current densities as a function of *η* (that is, log* j *~*η*) were recorded to probe useful kinetic metrics of studied catalysts, as shown in Figure 4(d). Tafel slope of ~45 mV per decade was measured for PdS_x_-Ni_3_S_4_, which is smaller than that of other catalysts except for the Pt/C benchmark ([Supplementary-material supplementary-material-1]), demonstrating its efficient HER kinetics. In acid, such a Tafel slope hints at a two-electron transfer process involved Volmer-Tafel mechanism [2]. We further studied the inherent HER activities of these catalysts by calculating their exchange current densities (*j*_0_; [Supplementary-material supplementary-material-1]). The obtained* j*_0_ of 5.62 × 10^−2^ mA cm^−2^ for PdS_x_-Ni_3_S_4_ makes it a remarkable HER catalyst that heads for the Pt/C benchmark ([Supplementary-material supplementary-material-1]). Moreover, the turnover frequency (TOF) of H_2_ molecules evolved per second was calculated to be 108 s^−1^ at -300 mV for the PdS_x_-Ni_3_S_4_, substantially exceeding the pure Ni_3_S_4_ with TOF of 3.2 s^−1^ (Figure 4(e)).

Additional evidence that PdS_x_-Ni_3_S_4_ heterocatalyst gives promoted HER reactivity was demonstrated with the double-layer capacitance (*C*_dl_), which is proportional to the effective electrochemically active surface area [50] (Figures 4(f), [Supplementary-material supplementary-material-1], and [Supplementary-material supplementary-material-1]). The measured large *C*_dl_ of 9.75 mF cm^−2^ for PdS_x_-Ni_3_S_4_ heterocatalyst implies its high exposure of catalytic active sites, comparing favorably with that of 1.71 mF cm^−2^ for pure Ni_3_S_4_. Electrochemical impedance spectroscopy (EIS) was next recorded at a *η* of 200 mV to probe the charge transfer resistance (*R*_ct_) for studied catalysts (Figure 4(g)). The measured *R*_ct_ of 3.1 Ohm for PdS_x_-Ni_3_S_4_ is considerably lower than that for pure Ni_3_S_4_ (537.2 Ohm), which indicates superior Faradaic process of PdS_x_-Ni_3_S_4_ heterocatalyst, in agreement with our work function measurements presented above (Figure 3(f)).

Our measurement of the superior HER activity on PdS_x_-Ni_3_S_4_ heteronanorods implies a long-range effect of the PdS_x_-Ni_3_S_4_ interface on the reactivity of Ni_3_S_4_ nanorods. We consider that the enhanced energetics do not originate exclusively from the localized nanoscale interface. Rather, remote Ni_3_S_4_ surface is activated resulting from the greatly modulated electronic structure discussed in Figure 3. Such pronounced modulation is unlikely to realize by the small population of accessible interfaces in PdS_x_-Ni_3_S_4_ heteronanorods. We further ascertain the enhancement that results from the long-range activation rather than the nanoscale interface by comparing the HER properties for the aforementioned size series of heteronanorods with Ni_3_S_4_ mean length of 15.0 nm, 25.1 nm, and 34.8 nm. We observe abrupt increase in HER activity for different sized heteronanorods relative to pure Ni_3_S_4_ nanorods (Figures 4(b)–4(g)), indicating substantial activation of Ni_3_S_4_ induced by PdS_x_. In Figures 4(b)–4(g), our electrochemical measurements also uncover an activity trend of PdS_x_-Ni_3_S_4_ heteronanorods (25.1 nm) > PdS_x_-Ni_3_S_4_ (34.8 nm) > PdS_x_-Ni_3_S_4_ (15.0 nm). This trend of experimental activities suggests that Ni_3_S_4_ nanorods are able to be activated up to ~25 nm away from the interface (Figure 4(h)). It is clear that, at the same mass loading of the PdS_x_-Ni_3_S_4_ heteronanorods, shorter Ni_3_S_4_ nanorods (15.0 nm) bring excess inactive PdS_x_ but longer Ni_3_S_4_ nanorods (34.8 nm) are mere partially activated. More proportional of inactive PdS_x_ (in shorter heteronanorods) and unactivated Ni_3_S_4_ (in longer heteronanorods) both lead to inferior activities. These results give conclusive experimental evidence that long-range activation enabled by interfacial interaction is indeed realized in the PdS_x_-Ni_3_S_4_ heterocatalyst.

### 2.4. Performance Stability

**Figure 5:**
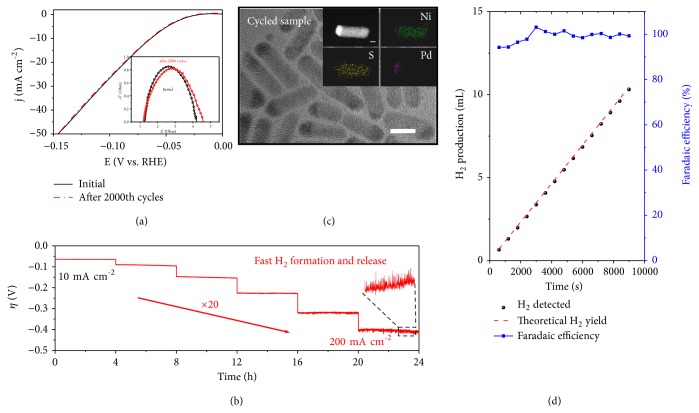
*Catalytic stability of PdS*_*x*_ *-Ni*_3_ *S*_4_ *heterocatalyst.* (a) HER polarization curves of PdS_x_-Ni_3_S_4_ catalyst before and after 2,000 potential cycles between -200 and 200 mV versus RHE. Inset shows the Nyquist plots before and after stability tests.* The potential for the Nyquist plot measurement was -0.204 V *versus* RHE.* (b) Multistep chronopotentiometric curve for the PdS_x_-Ni_3_S_4_ catalyst, showing that this new heterocatalyst runs robustly even at a high current density of 200 mA cm^−2^. (c) TEM image (scale bar: 10 nm) and STEM-EDX mapping (inset; scale bar: 5 nm) of PdS_x_-Ni_3_S_4_ heterocatalyst after 2,000 cycles of accelerated stability test, respectively. (d) Current efficiency for H_2_ evolution catalyzed by the PdS_x_-Ni_3_S_4_ catalyst, showing a Faradic efficiency close to 100%.

We now turn to assess the chemical and structural stability of the new PdS_x_-Ni_3_S_4_ heterocatalyst. In Figure 5(a) we show the long-term CV cycling data, which reveals only negligible decay after 2,000 cycles between -200 and 200 mV versus RHE. This observation is in agreement with our EIS measurements, where the Nyquist plots exhibit a mere 0.33 Ohm increase of *R*_ct_ after cycling (Inset in Figure 5(a)). We performed further stability test by running the HER on PdS_x_-Ni_3_S_4_ heterocatalyst under currents from 10 to 200 mA cm^−2^ continuously for 24 hours. No appreciable increase in *η* is seen in Figure 5(b), even at the high current density of 200 mA cm^−2^, underscoring its striking robustness. After electrolysis cycles, the catalyst was removed from carbon paper and characterized by TEM, EDS and elemental mapping, which show that the ‘dot-on-rod' structure is maintained with previous elemental distribution (Figures 5(c) and [Supplementary-material supplementary-material-1]). Furthermore, our XPS analysis reveals no obvious chemical state changes after stability test ([Supplementary-material supplementary-material-1]). The above results illustrate the remarkable performing stability of the new PdS_x_-Ni_3_S_4_ heterocatalysts, suggesting the potential electrode application. We finally detected the catalytic generation of H_2_ on PdS_x_-Ni_3_S_4_ electrode by gas chromatography, which is consistent with the theoretical value, corresponding to a Faradaic efficiency of ~100% (Figure 5(d)).

## 3. Discussion

Strong interfacial interaction that leads to enhanced catalytic properties was widely affirmed in metal-support (e.g., oxides) heterogeneous catalysts [5–14]. As mentioned above, such interaction involves charge transfer across the metal oxide interface, enabling surface modulation of supported metals, and, hence, their improved activities [9, 10]. This interaction is commonly thought to be localized within 1 nm around the interface region, owing to the intrinsic limit of charge transfer set by the support [5, 7]. Yet this charge transfer could be in principle regulated through tuning the structure and chemical properties of the support, as detailed in previous reports [6, 8]. An earlier research described that CO oxidation on CeO_2_-supported group VIII metals is localized, where the nanoscale perimeter atoms are active sites [8]. Intriguingly, Suchorski et al. have recently showed that ZrO_2_ (also Al_2_O_3_ and other oxides) supported Pd aggregates (50-200 *μ*m) enable high CO tolerance throughout the entire Pd particles owing to the metal oxide interaction effect, leading to remote activation of Pd up to thousands of nanometers [5]. Although current PdS_x_-Ni_3_S_4_ ‘dot-on-rod' structure somewhat differs from the conventional metal oxide heterogeneous catalysts, the substantially enhanced HER performances seen here are also likely the result of long-range activation caused by the PdS_x_-Ni_3_S_4_ interface based on the large electronic property changes of Ni_3_S_4_ nanorods that uncovered by multiple characterizations. Detailed explanations for this remote activation are still lacking and require further investigations.

In summary, we here demonstrate an unprecedented long-range activation of polydymite Ni_3_S_4_ nanorods due to the interfacial interaction created by nanoparticulate PdS_x_ terminations, which results in substantial HER efficiency gains. This strong impact on Ni_3_S_4_ rods with length up to 25 nm arises from the modified surface electronic structure based on various experimental investigations, somewhat analogous to a remote activation observed recently on Pd-oxides catalysts for CO oxidation [5]. We expect that such long-range effect from nanoscopic interfaces is not unique to Ni_3_S_4_, but bears general implications for other catalyst systems beyond metal chalcogenides. This work provides an unconventional pathway towards a wide range of materials whose performances are highly attractive for electrocatalysis.

## 4. Materials and Methods

### 4.1. Synthesis of PdS_X_-*Ni*_3_*S*_4_ Heteronanorods

In a typical procedure, 0.05 mmol of PdCl_2_, 0.2 mmol nickel (II) 2,4-pentanedionate (Ni(acac)_2_, Alfa Aesar, 99%), and 5 mL oleylamine (OAm) were loaded into a 25 mL three-necked flask under stirring. The mixture was heated under N_2_ atmosphere to 100°C and kept at this temperature for 30 mins. And then 0.5 mL 1-dodecanethiol was injected into the solution in sequence. After that the mixture solution was heated to 250°C at a heating rate of 10°C/min and incubated at this temperature for 30 min, generating a black solution. After cooling to room temperature, black precipitate was obtained by adding a large amount of ethanol into the colloidal solution and centrifugated at 10000 rpm for 5 mins. The precipitate was washed three times with excessive ethanol and redispersed in hexane.

### 4.2. Synthesis of Pure *Ni*_3_*S*_4_ Nanorods

Pure Ni_3_S_4_ nanorods were synthesized by similar procedures to that described for the synthesis of PdS_x_-Ni_3_S_4_ hybrid nanorods in the absence of 0.05 mmol of PdCl_2_.

### 4.3. Characterization

The samples were characterized by different analytic techniques. XRD was performed on a Philips X'Pert Pro Super X-ray diffractometer equipped with graphite-monochromatized Cu Ka radiation (*λ* = 1.54178 Å). Scanning electron microscope (SEM, Zeiss Supra 40) and JEOL 2010F(s) TEM were applied to investigate the size and morphology. The HRTEM images, EELS, SAED, and EDX elemental mappings were taken on JEMARM 200F Atomic Resolution Analytical Microscope with an acceleration voltage of 200 kV. XPS was performed by an X-ray photoelectron spectrometer (ESCALab MKII) with an excitation source of mg K*α* radiation (1253.6 eV). ICP data were obtained by an Optima 7300 DV instrument. Ultraviolet photoelectron spectroscopy was carried out at the BL11U beamline of National Synchrotron Radiation Laboratory in Hefei, China. The X-ray absorption spectra of Ni and S K-edges were obtained at the beamline 4B7A station of Beijing Synchrotron Radiation Facility (China).

### 4.4. Electrochemical Measurements

Electrochemical measurements were performed using a Multipotentiostat (IM6ex, ZAHNER elektrik, Germany). All measurements in 0.5 M H_2_SO_4_ were performed using a three-electrode cell. A graphite rod and Ag/AgCl (PINE, 3.5 M KCl) were used as counter and reference electrodes, respectively. 5 mg of catalyst powder was dispersed in 1 ml isopropanol with 20 *μ*l of Nafion solution (5 wt%, Sigma-Aldrich); then the mixture was ultrasonicated for at least 30 min to generate a homogeneous ink. Next, 200 *μ*l of the dispersion was transferred onto the 1 cm^2^ carbon fiber paper, leading to the catalyst loading ~1 mg cm^−2^. All the potentials in this study were referenced to Ag/AgCl (measured) or the reversible hydrogen electrode (RHE). Before the electrochemical measurement, the electrolyte (0.5 M H_2_SO_4_) was degassed by bubbling N_2_ for 30 min. The polarization curves were obtained by sweeping the potential from -0.7 to 0.2 V versus Ag/AgCl at room temperature with a sweep rate of 5 mV s^−1^. The accelerated stability tests were performed in N_2_-saturated 0.5 M H_2_SO_4_ at room temperature by potential cycling between -0.2 and 0.2 V versus RHE at a sweep rate of 100 mV s^−1^ for given number of cycles. At the end of each cycling, the resulting electrode was used for polarization curves. Chronoamperometric measurements of the catalysts on carbon fiber paper electrodes kept at a constant current density of 10 mA cm^−2^ in N_2_-saturated 0.5 M H_2_SO_4_. Multistep chronopotentiometric curve for the PdS_x_-Ni_3_S_4_ hybrid nanorods was tested with current density increasing from 10 to 200 mA cm^−2^. CV measurements taken with various scan rates (20, 40, 60 mV s^−1^, etc.) were conducted in static solution to estimate the double-layer capacitance by sweeping the potential across the nonfaradaic region 0.1-0.2 V versus RHE Electrochemical impedance spectroscopy measurement was performed when the working electrode was biased at a constant -0.40 V versus Ag/AgCl while sweeping the frequency from 100 kHz to 100 mHz with a 5 mV AC dither.

The values of TOF were calculated by assuming that every metal atom is involved in the catalysis (lower TOF limits were calculated):

(1) $$\begin{array}{c} TOF=\frac{j\times S}{2\times F\times n} \end{array}$$

Here, j (mA cm^−2^) is the measured current density, S is the geometric area of carbon paper, the number 2 means 2 electrons/mol of H_2_, F is Faraday constant (96485.3 C mol^−1^), and n is the moles of coated metal atom on the electrode calculated from the deposited catalysts.

## Data Availability

All data needed to evaluate the conclusions in the paper are present in the paper and the Supplementary Materials. Additional data related to this paper may be requested from the authors.

## Supplementary Materials

Supplementary Materials Figure S1: HRTEM characterization. Figure S2: HRTEM and FFT characterizations. Figure S3: EDS spectrum of the PdS_x_-Ni_3_S_4_ heteronanorods. Figure S4: STEM-EDX elemental mapping of a single PdS_x_-Ni_3_S_4_ heteronanorod. Figure S5: EDS line analysis of a typical PdS_x_-Ni_3_S_4_ heteronanorod. Figure S6: size characterizations. Figure S7: TEM images of the products synthesized by using different amount of 1-dodecanethiol. Figure S8: TEM images of products obtained at different temperature. Figure S9: TEM images of the products synthesized with different Pd:Ni radio. Figure S10: TEM images of the products synthesized at 250°C for different reaction time. Figure S11: TEM images of pure Ni_3_S_4_ nanorods. Figure S12: The SAED patterns of PdS_x_-Ni_3_S_4_ and pure Ni_3_S_4_ nanorods. Figure S13: Characterization of Pd precursor and pure PdS_x_ nanoparticles. Figure S14: XPS spectra analysis. Figure S15: FT-IR spectra of PdS_x_-Ni_3_S_4_ heteronanorods before and after acetic acid treatment. Figure S16: HER performance for the products obtained at different reaction time and the products obtained with different ratio of Pd:Ni. Figure S17: HER performance for the products obtained with different amount of C_12_SH and the products obtained at different temperatures. Figure S18: comparison of the onset potential required to start the HER on various Ni-based electrocatalysts. Figure S19: Tafel plot for the Pt/C (20 wt%) benchmark. Figure S20: exchange current density for different studied catalysts. Figure S21: capacitance measurement. Figure S22: Capacitance measurement. Figure S23: EDS spectrum of the PdS_x_-Ni_3_S_4_ heteronanorods after 2000 cyclic voltammetry cycles. Figure S24: XPS spectra for the PdS_x_-Ni_3_S_4_ heterocatalysts before and after 2000 potential cycles. Table S1: comparison of catalytic parameter of different Pt-free HER catalysts.
