# Supplementary material for: Synthesis of PdSx-Mediated Polydymite Heteronanorods and Their Long-Range Activation for Enhanced Water Electroreduction
Source: Research (Wash D C). 2019 Aug 18;2019:8078549. doi: 10.34133/2019/8078549 (PMC6750066; doi:10.34133/2019/8078549)
Supplement: Supplementary Materials — Figure S1: HRTEM characterization. Figure S2: HRTEM and FFT characterizations. Figure S3: EDS spectrum of the PdSx-Ni3S4 heteronanorods. Figure S4: STEM-EDX elemental mapping of a single PdSx-Ni3S4 heteronanorod. Figure S5: EDS line analysis of a typical PdSx-Ni3S4 heteronanorod. Figure S6: size characterizations. Figure S7: TEM images of the products synthesized by using different amount of 1-dodecanethiol. Figure S8: TEM images of products obtained at different temperature. Figure S9: TEM images of the products synthesized with different Pd:Ni radio. Figure S10: TEM images of the products synthesized at 250°C for different reaction time. Figure S11: TEM images of pure Ni3S4 nanorods. Figure S12: The SAED patterns of PdSx-Ni3S4 and pure Ni3S4 nanorods. Figure S13: Characterization of Pd precursor and pure PdSx nanoparticles. Figure S14: XPS spectra analysis. Figure S15: FT-IR spectra of PdSx-Ni3S4 heteronanorods before and after acetic acid treatment. Figure S16: HER performance for the products obtained at different reaction time and the products obtained with different ratio of Pd:Ni. Figure S17: HER performance for the products obtained with different amount of C12SH and the products obtained at different temperatures. Figure S18: comparison of the onset potential required to start the HER on various Ni-based electrocatalysts. Figure S19: Tafel plot for the Pt/C (20 wt%) benchmark. Figure S20: exchange current density for different studied catalysts. Figure S21: capacitance measurement. Figure S22: Capacitance measurement. Figure S23: EDS spectrum of the PdSx-Ni3S4 heteronanorods after 2000 cyclic voltammetry cycles. Figure S24: XPS spectra for the PdSx-Ni3S4 heterocatalysts before and after 2000 potential cycles. Table S1: comparison of catalytic parameter of different Pt-free HER catalysts. [file 8078549.f1.pdf]

Supplementary Materials

Synthesis of PdS_x-Mediated Polydymite Heteronanorods and Their Long-Range Activation for Enhanced Water Electroreduction

Qiang Gao,[1,†] Rui Wu,[1,†] Yang Liu,[1] Ya-Rong Zheng,[1] Yi Li,[1] Li-Mei Shang,[1] Yi-Ming Ju,[1] Chao Gu,[1] Xu-Sheng Zheng,[2] Jian-Wei Liu,[1] Jun-Fa Zhu,[2] Min-Rui Gao,[1,*] and Shu-Hong Yu[1,2*]

[1]Division of Nanomaterials & Chemistry, Hefei National Laboratory for Physical Sciences at the Microscale, CAS Center for Excellence in Nanoscience, Hefei Science Center of CAS, Collaborative Innovation Center of Suzhou Nano Science and Technology, Department of Chemistry, University of Science and Technology of China, Hefei 230026, China

[2]National Synchrotron Radiation Laboratory, University of Science and Technology of China, Hefei 230026, China

[†]These authors contributed equally to this work.

[*]Corresponding author: mgao@ustc.edu.cn; shyu@ustc.edu.cn.

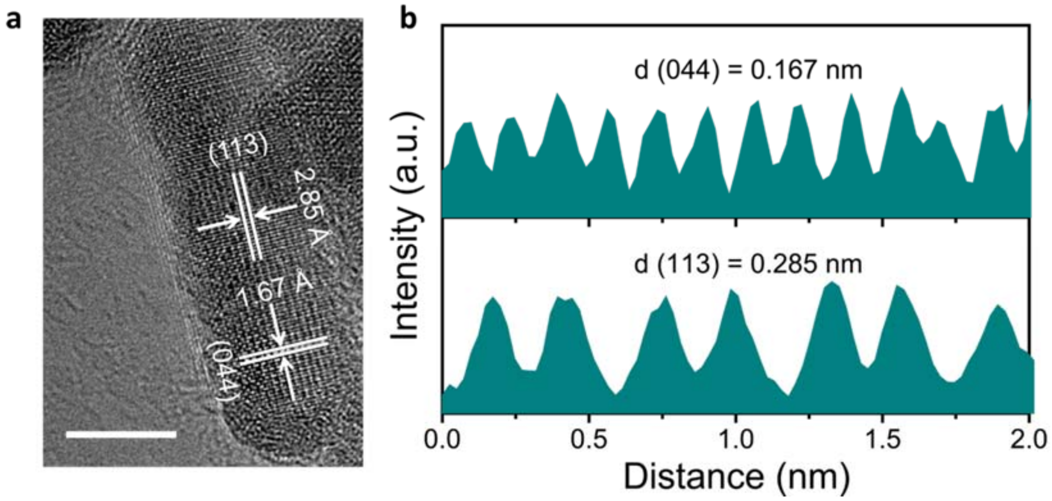

Figure S1. (a) HRTEM image of a single PdS_x-Ni_3S_4 heteronanorod. Scale bar, 5 nm. (b) The integrated pixel intensities of spacings along (044) and (113) facets.

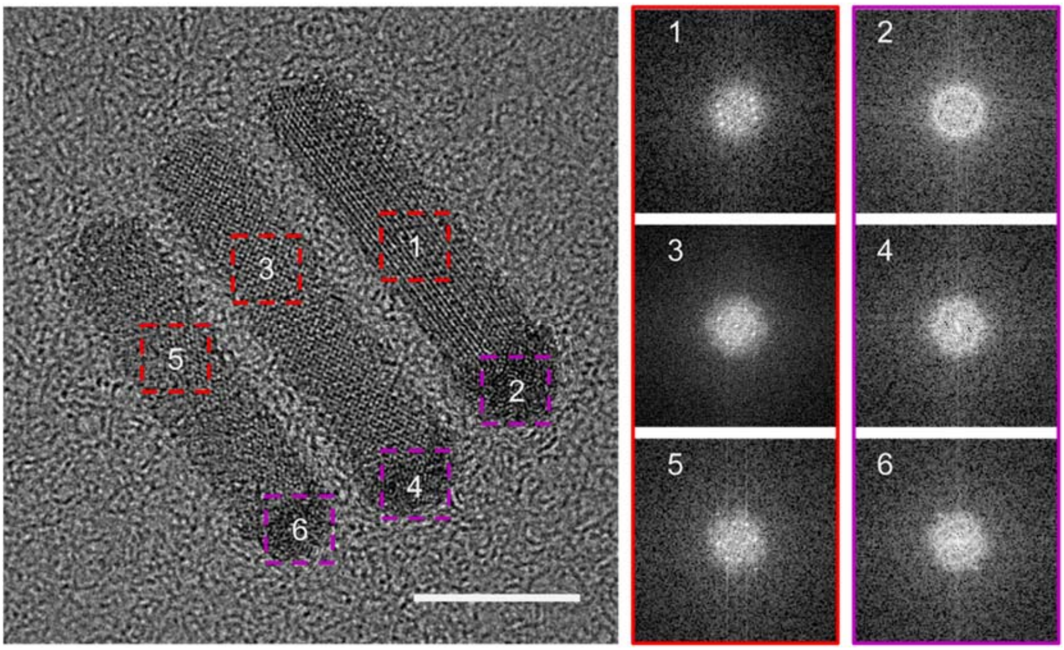

Figure S2. HRTEM image of three PdS_x-Ni_3S_4 heteronanorods and corresponding fast Fourier transform (FFT) patterns: the rods (1, 3, 5) and the dots (2, 4, 6). Scale bar, 10 nm. These characterizations clear reveal the ‘dot-on-rod’ heterostructure that corresponds to PdS_x (amorphous) on Ni_3S_4 (crystalline).

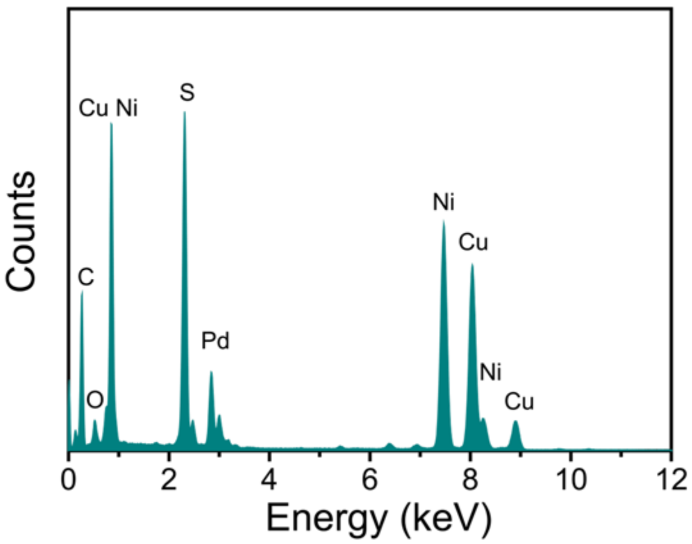

Figure S3. EDS spectrum of the as-synthesized PdS_x-Ni_3S_4 heteronanorods.

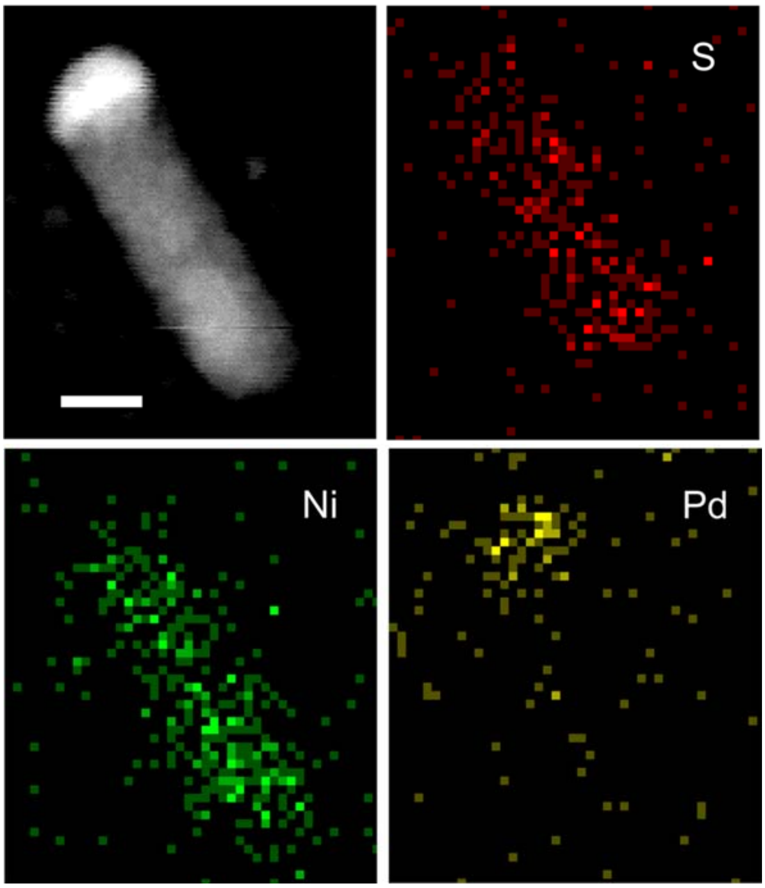

Figure S4. Additional STEM-EDX elemental mapping of a single PdS_x-Ni_3S_4 heteronanorod. Scale bar, 5 nm.

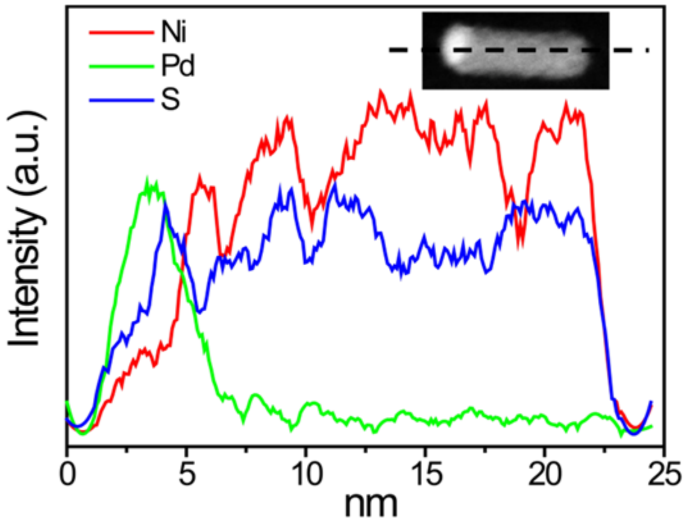

Figure S5. EDS line analysis of a typical PdS_x-Ni_3S_4 heteronanorod, which shows clear the Pd-rich dot and Ni-rich rod, while the S enriches in the whole structure.

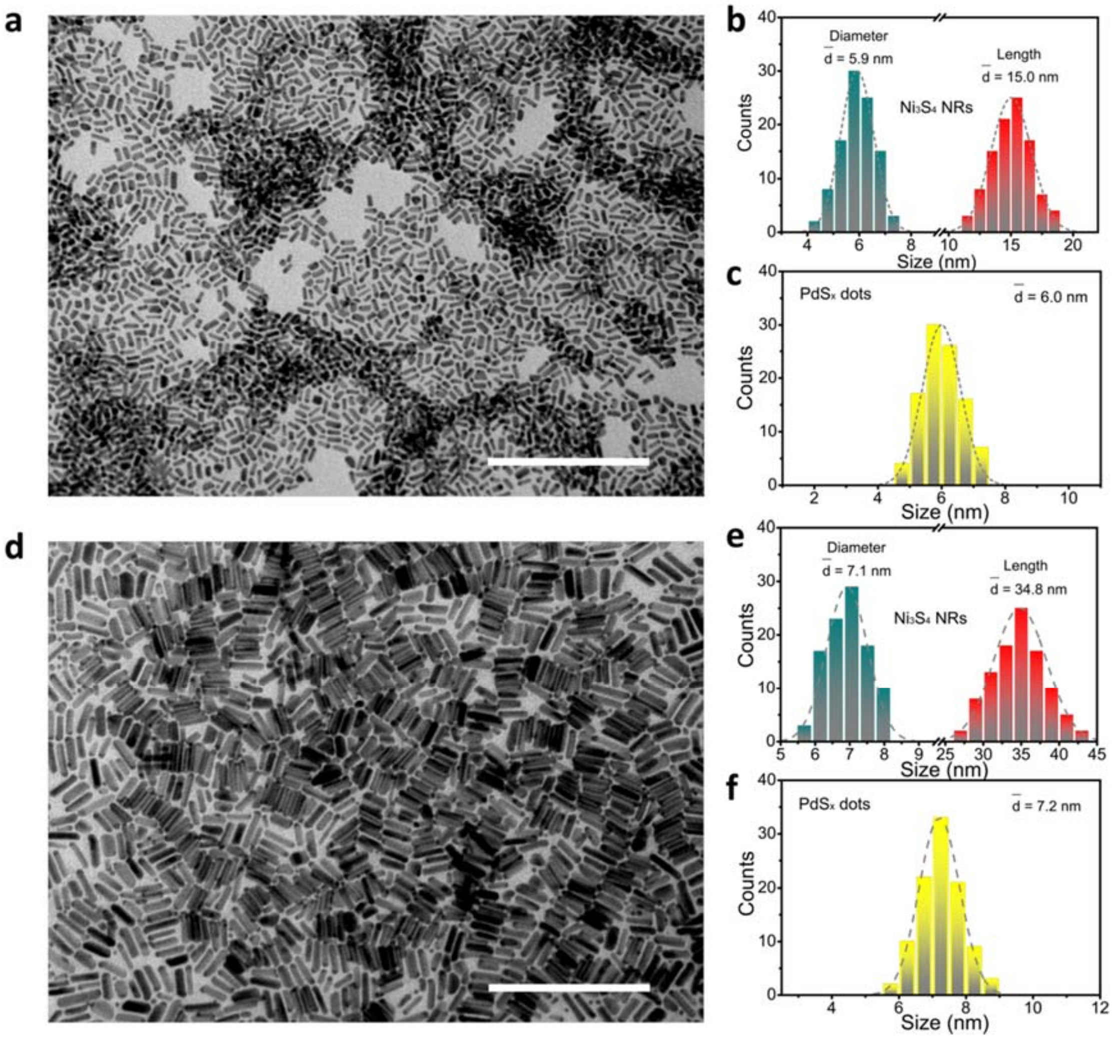

Figure S6. (a) TEM image of the product synthesized with the addition of 0.25 mL 1-dodecanethiol. (b and c) The corresponding histograms show the mean size for Ni_3S_4 nanorods and PdS_x dots in the heteronanords, respectively. (d) TEM image of the product synthesized with the addition of 0.6 mL 1-dodecanethiol. (e and f) The corresponding histograms show the mean size for Ni_3S_4 nanorods and PdS_x dots in the heteronanords, respectively. Scale bars, 200 nm.

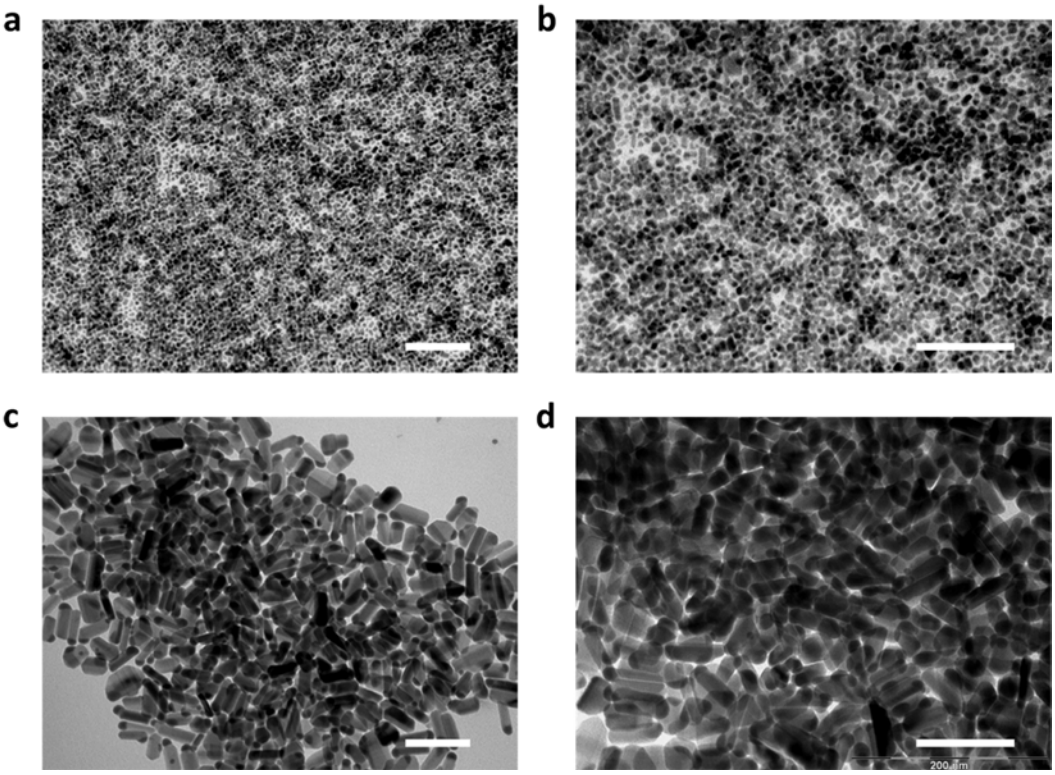

Figure S7. (a and b) TEM images of the products synthesized with addition of 0.1 mL 1-dodecanethiol. (c and d) TEM images of the products synthesized with addition of 0.75 mL 1-dodecanethiol. Scale bars, 100 nm.

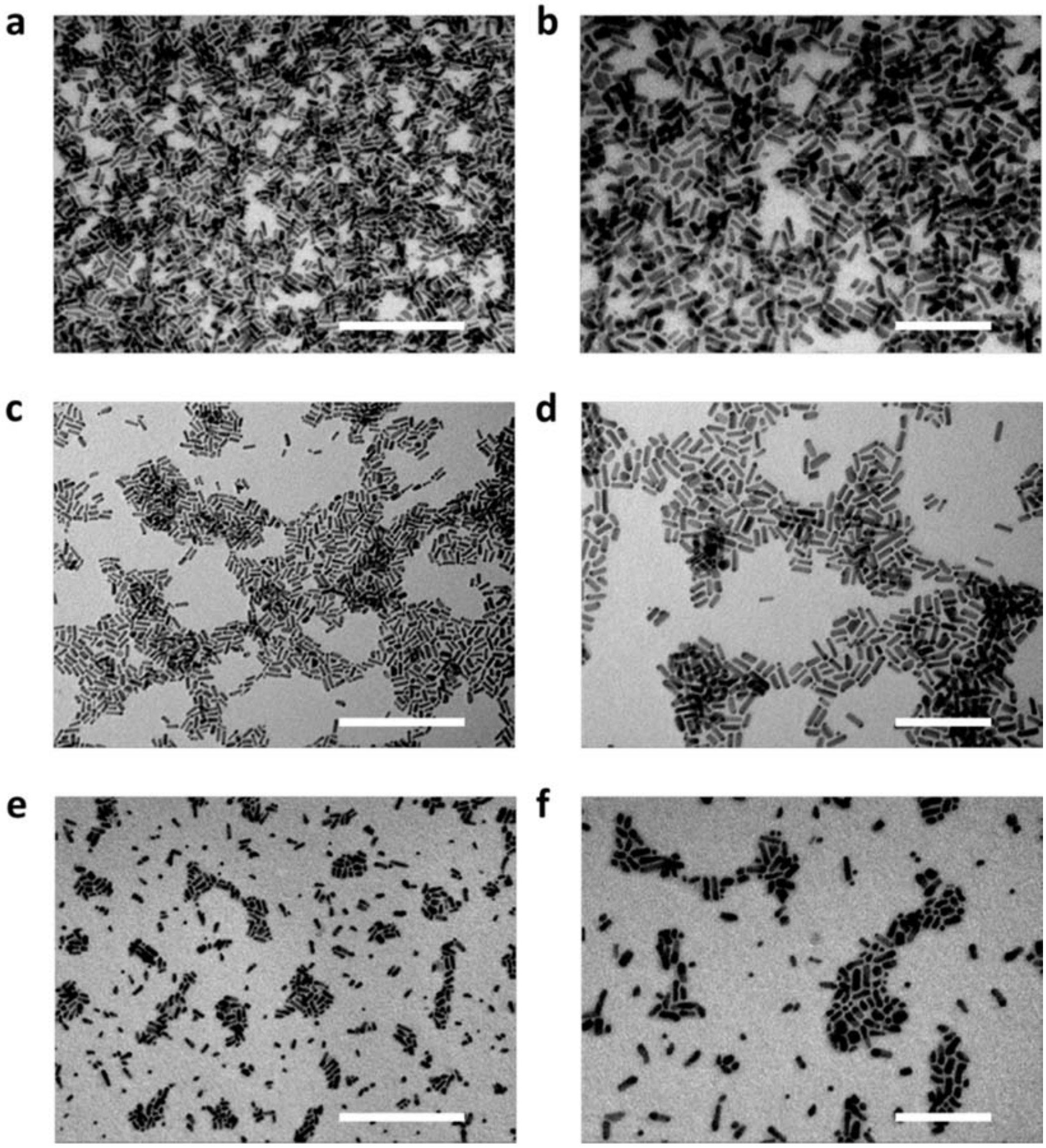

Figure S8. TEM images of products obtained at different temperature: (a and b) 230 ºC, (c and d) 250 ºC, (e and f) 270 ºC. Scale bars: 200 nm for (a), (c) ,(e) and 100 nm for(b), (d), (f).

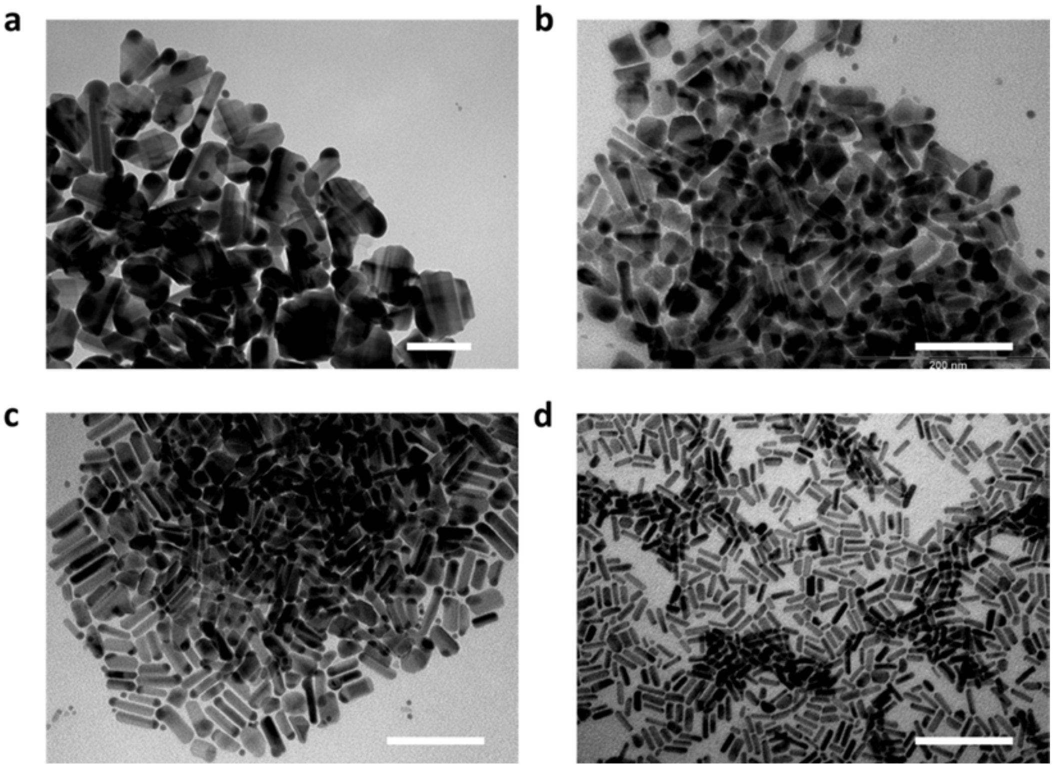

Figure S9. TEM images of the products synthesized with different Pd:Ni radio: (a) 2:1, (b) 1:1, (c) 1:2 and (d) 1:6. Scale bars, 100 nm.

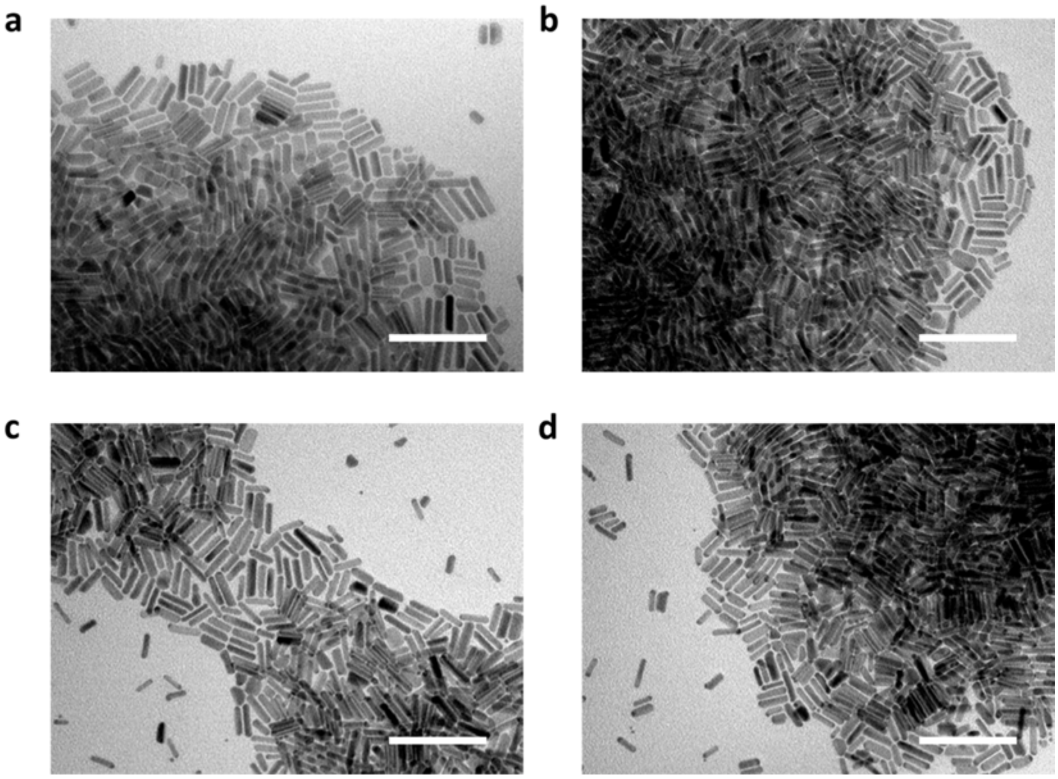

Figure S10. TEM images of the products synthesized at 250 ºC for different reaction times: (a) 0 min, (b) 5 min, (c) 10 min and (d) 20 min. Scale bars, 100 nm.

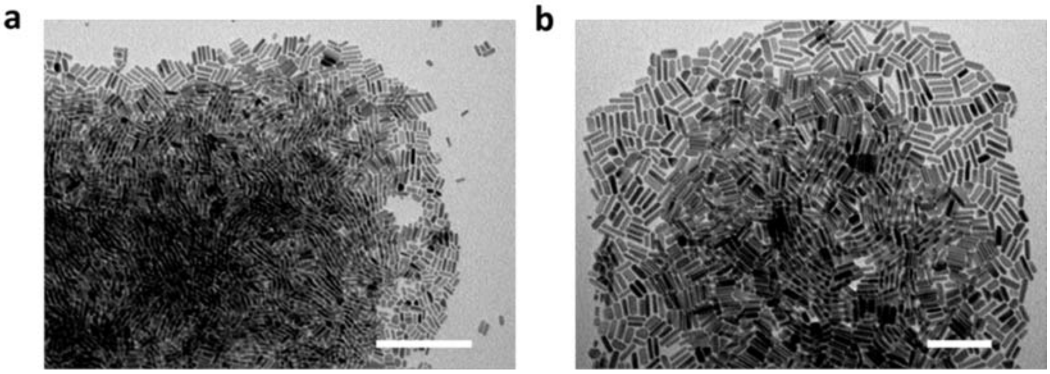

Figure S11. (a and b) Low and high magnification TEM images of pure Ni_3S_4 nanorods, respectively. Scale bars, 200 and 100 nm, respectively.

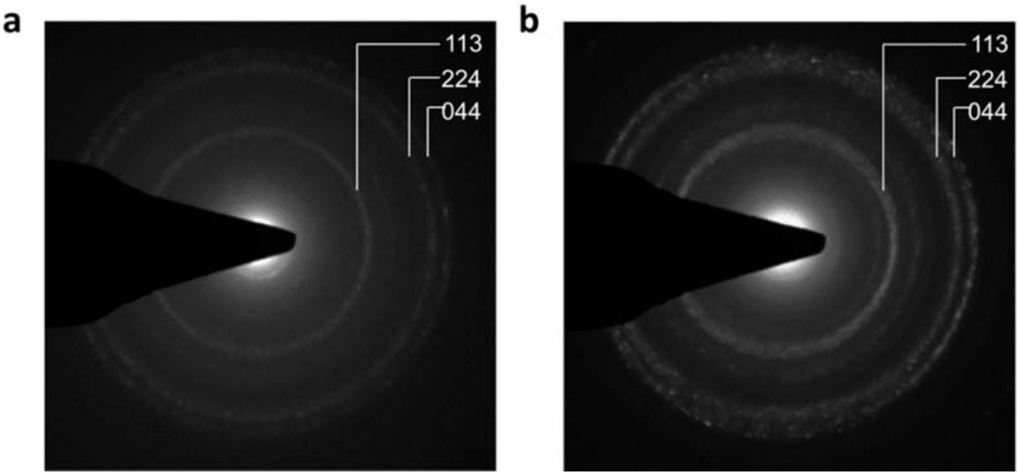

Figure S12. (a) The SAED pattern of PdS_x-Ni_3S_4, where the concentric rings are attributed to cubic Ni_3S_4 (113), (224) and (044), no crystalline PdS_x is detected. (b) The SAED pattern of pure Ni_3S_4, consistent with the result shown in (a).

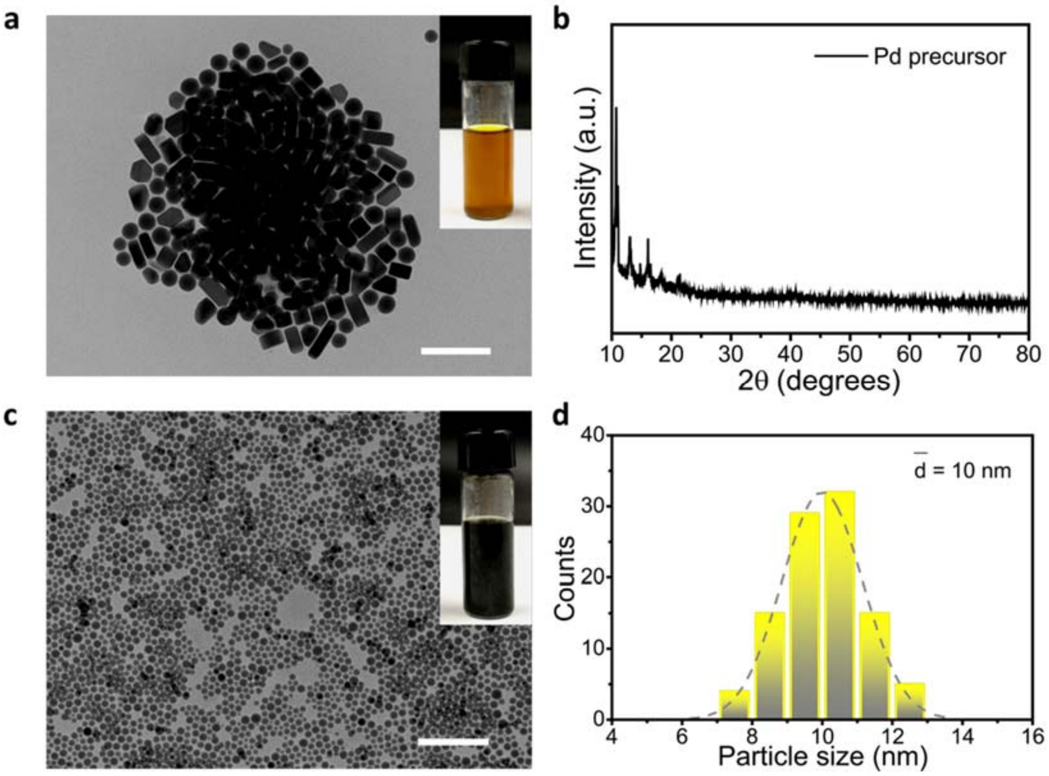

Figure S13. Characterization of Pd precursor and pure PdS_x nanoparticles. (a) TEM image of Pd precursor obtained at 250 ºC. Inset shows the photograph of corresponding Pd precursor dispersed in hexane. (b) XRD pattern of the Pd precursor obtained at 250 ºC. (c) TEM image of PdS_x nanoparticles obtained at 300 ºC. Inset shows the photograph of corresponding PdS_x nanoparticles dispersed in hexane. (d) The corresponding histogram of PdS_x nanoparticles, showing that the mean particle size is about 10 nm. Scale bars, 100 nm.

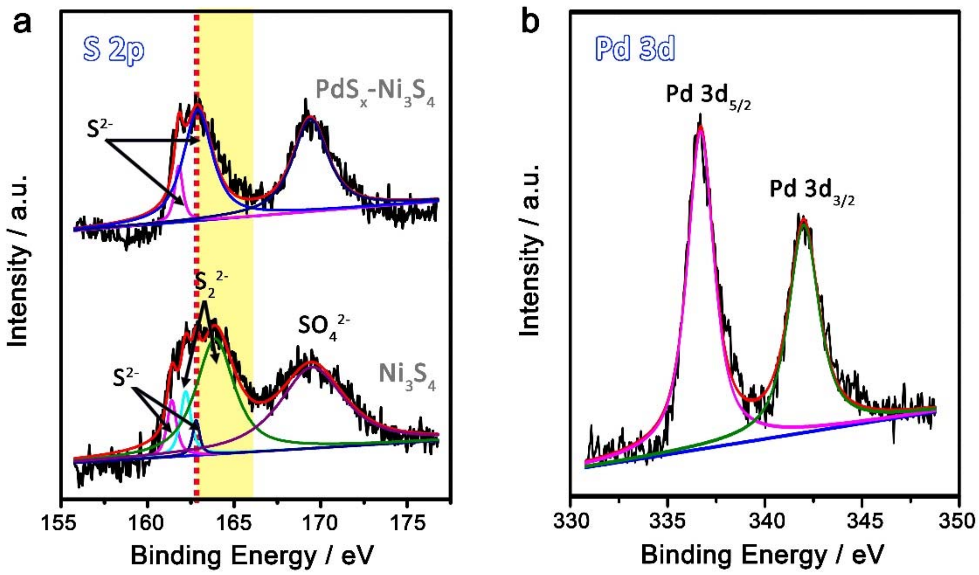

Figure S14. XPS spectra analysis. (a) S 2p XPS spectra for pure Ni_3S_4 and PdS_x-Ni_3S_4. (b) Pd 3d XPS spectrum for PdS_x-Ni_3S_4 heteronanorods.

The significant decrease of S_2^{2-} ion for PdS_x-Ni_3S_4 leads to numerous sulfur vacancies, which might promote the reactivity of exposed Ni sites and thus the HER activity. The participation of conductive PdS_x not only helps to increase the electric conductivity of Ni_3S_4, but, more importantly, creates favorable electronic interactions across the PdS_x-Ni_3S_4 interface, offering electron-rich Ni_3S_4 remote domains with modified structures that facilitate HER process.

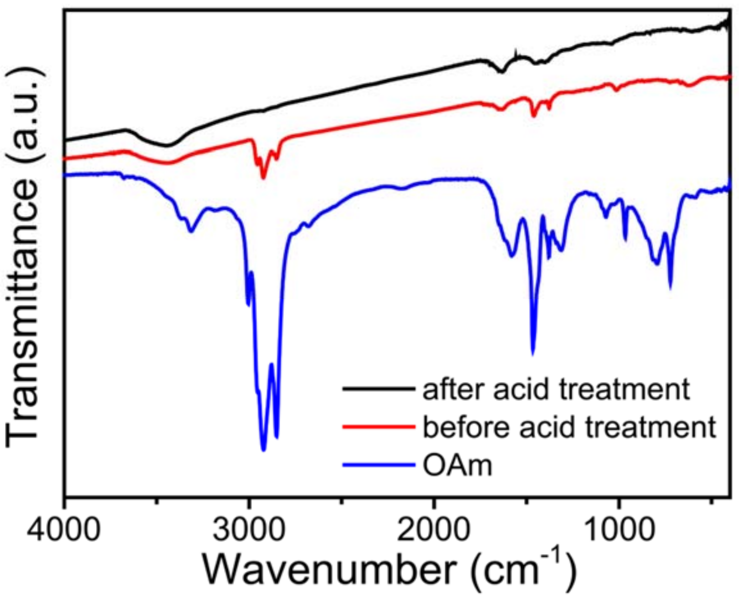

Figure S15. FT-IR spectra of PdS_x-Ni_3S_4 heteronanorods before and after acetic acid treatment, showing clearly that the surface OAm were thoroughly removed.

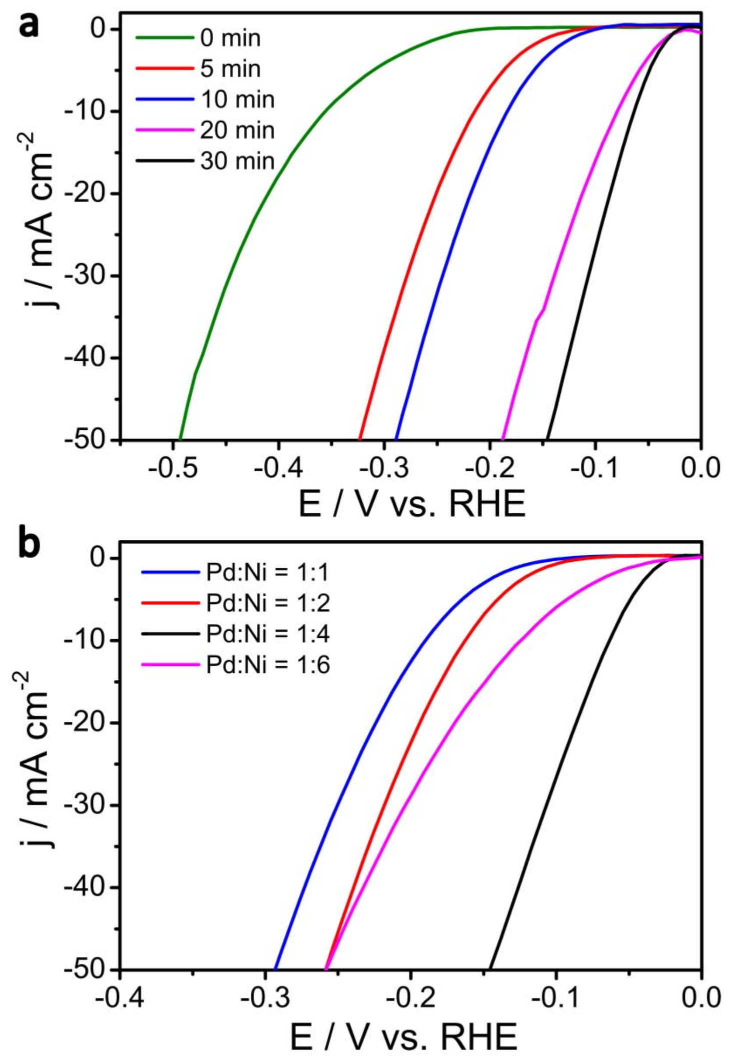

Figure S16. (a) HER polarization curves for the products obtained at different reaction times. (b) HER polarization curves for the products obtained with different ratio of Pd:Ni. All the measurements were performed in N_2-purged 0.5 M H_2SO_4. Catalyst loading: ~1 mg cm^{-2}. Sweep rate: 5 mV s^{-1}.

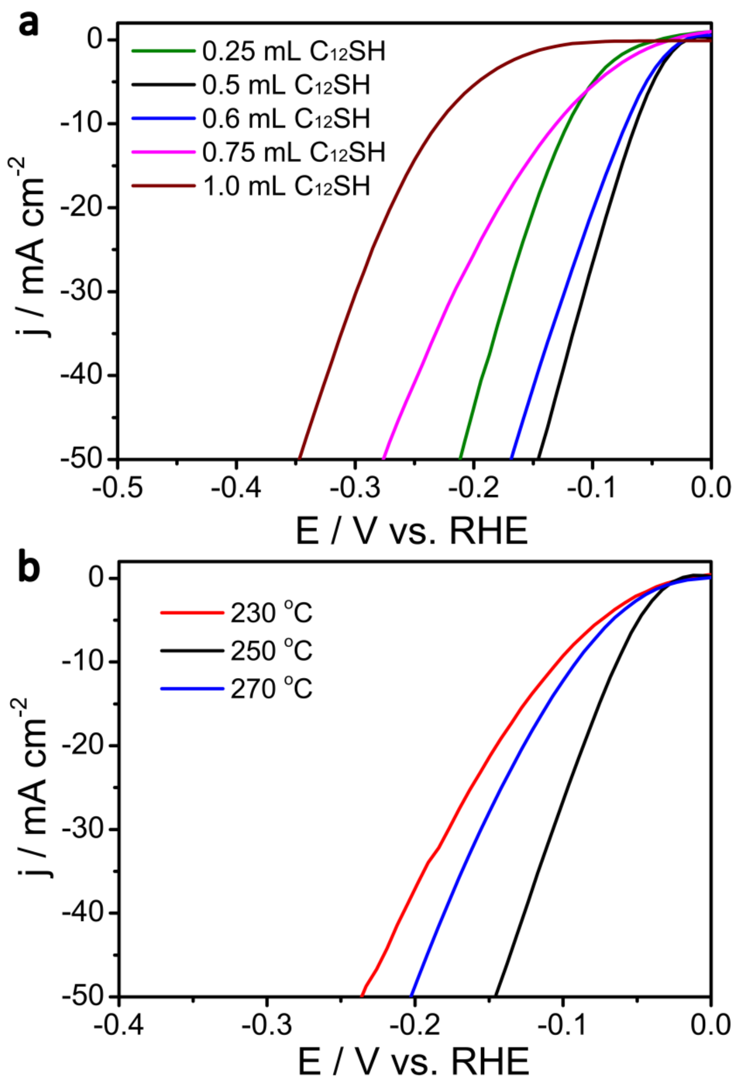

Figure S17. (a) HER polarization curves for the products obtained with different amount of $C_{12}SH$ during the synthesis. (b) HER polarization curves for the products obtained at different temperatures. All the measurements were performed in N_2-purged 0.5 M H_2SO_4. Catalyst loading: ~1 mg cm^{-2}. Sweep rate: 5 mV s^{-1}.

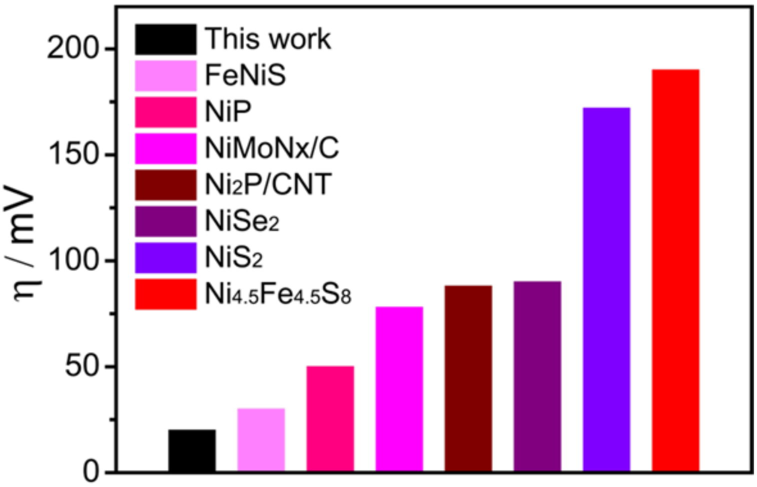

Figure S18. Comparison of the onset potential required to start the HER on various Ni-based electrocatalysts.

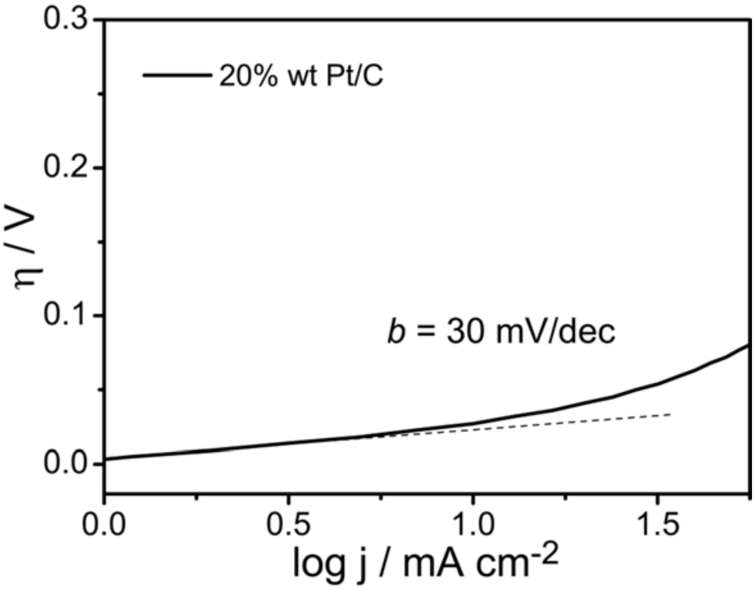

Figure S19. Tafel plot for the Pt/C (20 wt%) benchmark.

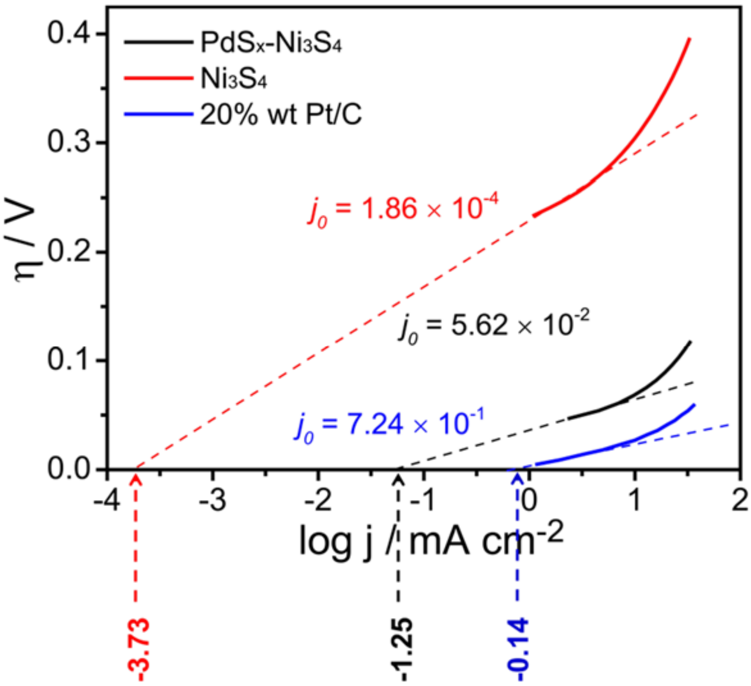

Figure S20. Calculated exchange current density for different studied catalysts in the work by using the extrapolation methods.

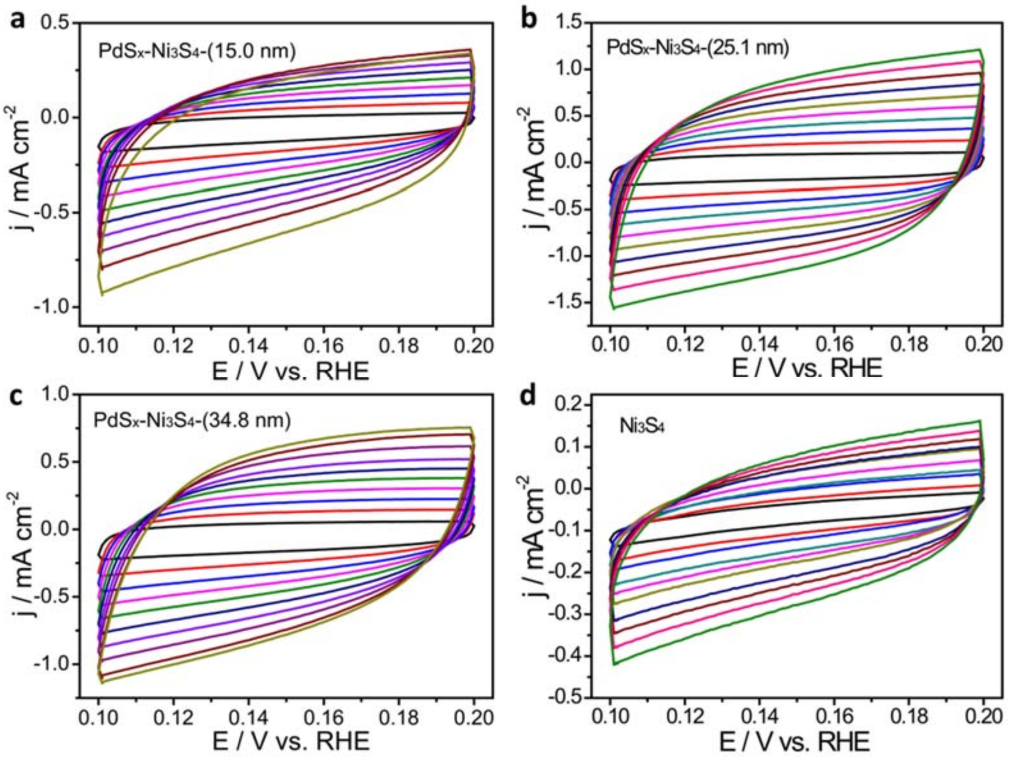

Figure S21. Cyclic voltammograms in the region of 0.1-0.2 V versus RHE for different studied samples: (a) 15.0 nm PdS_x-Ni_3S_4 heteronanorods, (b) 25.1 nm PdS_x-Ni_3S_4 heteronanorods, (c) 34.8 nm PdS_x-Ni_3S_4 heteronanorods and (d) pure Ni_3S_4 nanorods.

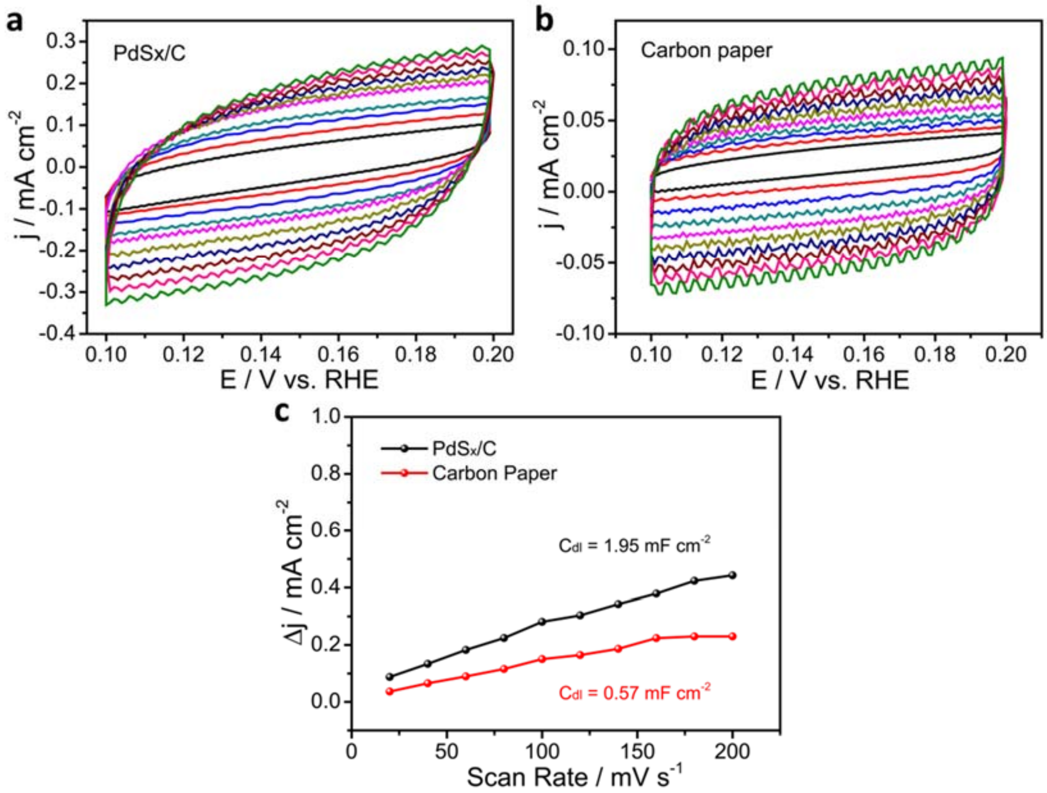

Figure S22. Cyclic voltammograms in the region of 0.1-0.2 V versus RHE for different materials: (a) carbon black supported PdS_x nanoparticles and (b) bare carbon paper. (c) Plots showing the extraction of the C_{dl} for different studied samples.

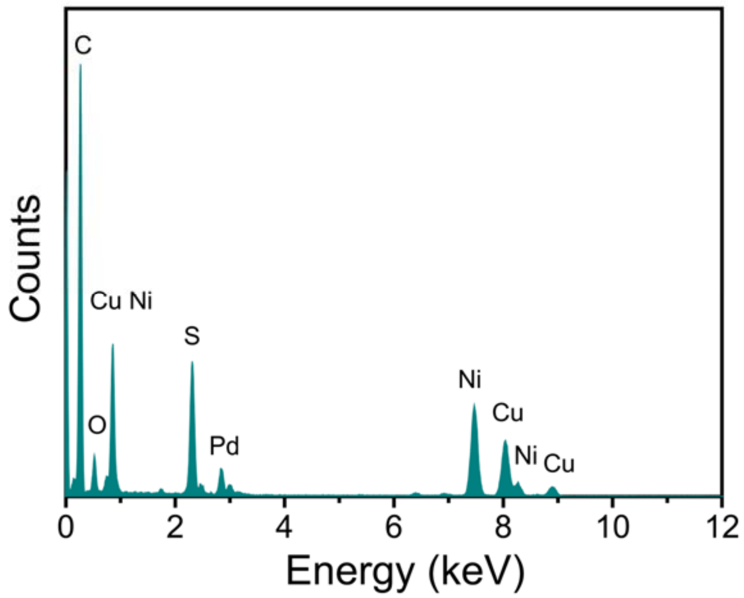

Figure S23. EDS spectrum of the PdS_x-Ni_3S_4 heteronanorods after 2000 cyclic voltammetry cycles.

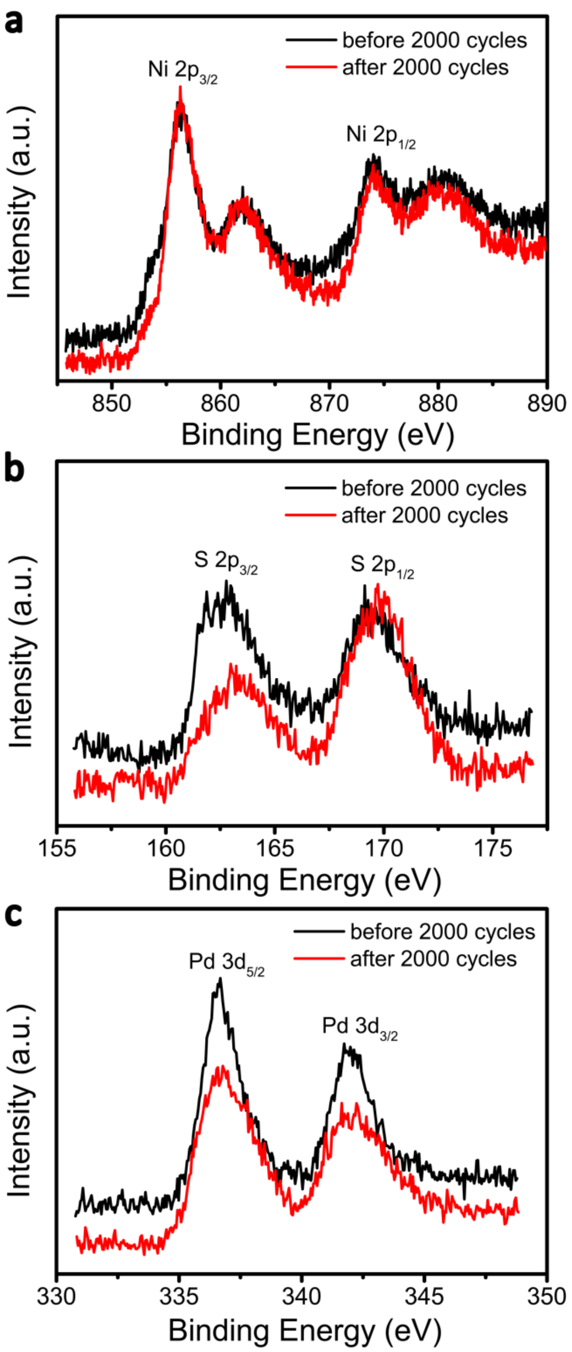

Figure S24. (a) Ni 2p, (b) S 2p and (c) Pd 3d XPS spectra for the PdS_x-Ni_3S_4 heterocatalysts before and after 2000 potential cycles. These data reveal that no obvious chemical state changes after the stability test, demonstrating the good chemical stability of the new PdS_x-Ni_3S_4 heteronanorod catalyst.

Table S1. Comparison of catalytic parameter of different Pt-free HER catalysts.

Catalysts	Onsetpotential (mV vs. RHE)	η @ j = 10 mA cm^{-2} (mV vs. RHE)	Tafel slope (mV dec^{-1})	j_0 (mA cm^{-2})	Ref.
PdS_x-Ni_3S_4	-20	-63	45	5.62×10^{-2}	This work
Ni_3S_4	-120	-304	90	1.86×10^{-4}	This work
Pt/C	0	-48	30	0.724	This work
NiS_2 film	N/A	N/A	41.6	1.4×10^{-5}	[1]
$NiSe_2$ film	N/A	N/A	56.9	7.5×10^{-4}	[1]
CoS_2 film	N/A	N/A	44.6	5.4×10^{-5}	[1]
$CoSe_2$ film	N/A	N/A	42.4	7.5×10^{-5}	[1]
CoS_2 Nanowire array	-75	-145	51.6	1.51×10^{-3}	[2]
β-NiS Nanosheets	N/A	-202	N/A	N/A	[3]
β-FeNiS Nanosheets	N/A	-117	48	1.4×10^{-2}	[3]
α-FeNiS Nanosheets	N/A	-105	40	2.0×10^{-2}	[3]
$NiSe_2$ porous nanosheets	-90	-135	37.3	6.46×10^{-3}	[4]
1T MoS_2 sheets	*ca.* -135	*ca.* -187	43	N/A	[5]
Interlayer-expanded MoS_2	-103	-149	49	9.62×10^{-3}	[6]
MoS_2/$CoSe_2$ hybrid	-11	-68	45	7.3×10^{-2}	[7]
MoS_2/rGO	-100	-150	41	N/A	[8]
MoS_2/N-doped CNT	-75	-110	40	N/A	[9]
$MoSe_2$/carbon paper	N/A	-250	59.8	3.8×10^{-4}	[10]
Mo_2C	-151	-214	56	1.3×10^{-3}	[11]
MoB	-155	-212	55	1.4×10^{-3}	[11]
$NiMoN_x$/C	-78	-152	35.9	0.24	[12]
$Ni_{4.5}Fe_{4.5}S_8$	*ca.* -50	*ca.* -110	46	3.3×10^{-2}	[13]
Ni_2P Nanoparticles	*ca.* -50	*ca.* -110	46	3.3×10^{-2}	[14]
Ni_2P /CNT	-88	-124	53	5.37×10^{-2}	[15]
CoP (on Ti)	N/A	*ca.* -72	50	0.14	[16]
CoP/CNT	-40	-122	N/A	0.13	[17]
CoPS	*ca.* -10	-48	56	0.984	[18]

References

[1] D. Kong, J. J. Cha, H. Wang, H. R. Lee, and Y. Cui, "First-row transition metal dichalcogenide catalysts for hydrogen evolution reaction," *Energy & Environmental Science,* vol. 6, no. 12, pp. 3553-3558, 2013.

[2] M. S. Faber, R. Dziedzic, M. A. Lukowski, N. S. Kaiser, Q. Ding, and S. Jin, "High-Performance Electrocatalysis Using Metallic Cobalt Pyrite (CoS_2) Micro- and Nanostructures," *Journal of the American Chemical Society,* vol. 136, no. 28, pp. 10053-10061, 2014.

[3] X. Long, G. Li, Z. Wang et al., "Metallic Iron–Nickel Sulfide Ultrathin Nanosheets As a Highly Active Electrocatalyst for Hydrogen Evolution Reaction in Acidic Media," *Journal of the American Chemical Society,* vol. 137, no. 37, pp. 11900-11903, 2015.

[4] H. Liang, L. Li, F. Meng et al., "Porous Two-Dimensional Nanosheets Converted from Layered Double Hydroxides and Their Applications in Electrocatalytic Water Splitting," *Chemistry of Materials,* vol. 27, no. 16, pp. 5702-5711, 2015.

[5] M. A. Lukowski, A. S. Daniel, F. Meng, A. Forticaux, L. Li, and S. Jin, "Enhanced Hydrogen Evolution Catalysis from Chemically Exfoliated Metallic MoS_2 Nanosheets," *Journal of the American Chemical Society,* vol. 135, no. 28, pp. 10274-10277, 2013.

[6] M. R. Gao, M. K. Y. Chan, and Y. G. Sun, "Edge-terminated molybdenum disulfide with a 9.4-angstrom interlayer spacing for electrochemical hydrogen production," *Nature Communications,* vol. 6, no. pp. 2015.

[7] M.-R. Gao, J.-X. Liang, Y.-R. Zheng et al., "An efficient molybdenum disulfide/cobalt diselenide hybrid catalyst for electrochemical hydrogen generation," *Nature Communications,* vol. 6, no. pp. 2015.

[8] Y. Li, H. Wang, L. Xie, Y. Liang, G. Hong, and H. Dai, "MoS_2 Nanoparticles Grown on Graphene: An Advanced Catalyst for the Hydrogen Evolution Reaction," *Journal of the American Chemical Society,* vol. 133, no. 19, pp. 7296-7299, 2011.

[9] D. J. Li, U. N. Maiti, J. Lim et al., "Molybdenum Sulfide/N-Doped CNT Forest Hybrid Catalysts for High-Performance Hydrogen Evolution Reaction," *Nano letters,* vol. 14, no. 3, pp. 1228-1233, 2014.

[10] D. Kong, H. Wang, J. J. Cha et al., "Synthesis of MoS_2 and $MoSe_2$ Films with Vertically Aligned Layers," *Nano letters,* vol. 13, no. 3, pp. 1341-1347, 2013.

[11] H. Vrubel, and X. Hu, "Molybdenum Boride and Carbide Catalyze Hydrogen Evolution in both Acidic and Basic Solutions," *Angewandte Chemie-International Edition,* vol. 124, no. 51, pp. 12875-12878, 2012.

[12] W.-F. Chen, K. Sasaki, C. Ma et al., "Hydrogen-Evolution Catalysts Based on Non-Noble Metal Nickel–Molybdenum Nitride Nanosheets," *Angewandte Chemie International Edition,* vol. 51, no. 25, pp. 6131-6135, 2012.

[13] B. Konkena, K. junge Puring, I. Sinev et al., "Pentlandite rocks as sustainable and stable efficient electrocatalysts for hydrogen generation," *Nature Communications,* vol. 7, no. pp. 12269, 2016.

[14] E. J. Popczun, J. R. McKone, C. G. Read et al., "Nanostructured Nickel Phosphide as an Electrocatalyst for the Hydrogen Evolution Reaction," *Journal of the American Chemical Society,* vol. 135, no. 25, pp. 9267-9270, 2013.

[15] Y. Pan, W. Hu, D. Liu, Y. Liu, and C. Liu, "Carbon nanotubes decorated with nickel phosphide nanoparticles as efficient nanohybrid electrocatalysts for the hydrogen evolution reaction," *Journal of Materials Chemistry A,* vol. 3, no. 24, pp. 13087-13094, 2015.
[16] E. J. Popczun, C. G. Read, C. W. Roske, N. S. Lewis, and R. E. Schaak, "Highly Active Electrocatalysis of the Hydrogen Evolution Reaction by Cobalt Phosphide Nanoparticles," *Angewandte Chemie International Edition,* vol. 53, no. 21, pp. 5427-5430, 2014.
[17] Q. Liu, J. Tian, W. Cui et al., "Carbon Nanotubes Decorated with CoP Nanocrystals: A Highly Active Non-Noble-Metal Nanohybrid Electrocatalyst for Hydrogen Evolution," *Angewandte Chemie International Edition,* vol. 53, no. 26, pp. 6710-6714, 2014.
[18] M. Caban-Acevedo, M. L. Stone, J. R. Schmidt et al., "Efficient hydrogen evolution catalysis using ternary pyrite-type cobalt phosphosulphide," *Nature Materials,* vol. 14, no. 12, pp. 1245-1251, 2015.
